# Infection with *Borrelia afzelii* and manipulation of the egg surface microbiota have no effect on the fitness of immature *Ixodes ricinus* ticks

**DOI:** 10.1038/s41598-021-90177-8

**Published:** 2021-05-21

**Authors:** Georgia Hurry, Elodie Maluenda, Anouk Sarr, Alessandro Belli, Phineas T. Hamilton, Olivier Duron, Olivier Plantard, Maarten J. Voordouw

**Affiliations:** 1 Department of Veterinary Microbiology, Western College of Veterinary Medicine, University of Saskatchewan, Saskatoon, Canada; 2 Laboratory of Ecology and Evolution of Parasites, Institute of Biology, University of Neuchâtel, Neuchâtel, Switzerland; 3 Laboratory of Ecology and Epidemiology of Parasites, Institute of Biology, University of Neuchâtel, Neuchâtel, Switzerland; 4 Deeley Research Centre, BC Cancer, Victoria, BC Canada; 5 Centre of Research in Ecology and Evolution of Diseases (CREES), Montpellier, France; 6 MIVEGEC (Maladies Infectieuses et Vecteurs: Ecologie, Génétique, Evolution et Contrôle), University of Montpellier (UM)-Centre National de la Recherche Scientifique (CNRS)-Institut pour la Recherche et le Développement (IRD), Montpellier, France; 7 INRAE, Oniris, BIOEPAR, Nantes, France

**Keywords:** Microbial ecology, Infectious-disease epidemiology, Symbiosis, Entomology, Pathogens

## Abstract

Arthropod vectors carry vector-borne pathogens that cause infectious disease in vertebrate hosts, and arthropod-associated microbiota, which consists of non-pathogenic microorganisms. Vector-borne pathogens and the microbiota can both influence the fitness of their arthropod vectors, and hence the epidemiology of vector-borne diseases. The bacterium *Borrelia afzelii*, which causes Lyme borreliosis in Europe, is transmitted among vertebrate reservoir hosts by *Ixodes ricinus* ticks, which also harbour a diverse microbiota of non-pathogenic bacteria. The purpose of this controlled study was to test whether *B. afzelii* and the tick-associated microbiota influence the fitness of *I. ricinus*. Eggs obtained from field-collected adult female ticks were surface sterilized (with bleach and ethanol), which reduced the abundance of the bacterial microbiota in the hatched *I. ricinus* larvae by 28-fold compared to larvae that hatched from control eggs washed with water. The dysbiosed and control larvae were subsequently fed on *B. afzelii*-infected or uninfected control mice, and the engorged larvae were left to moult into nymphs under laboratory conditions. *I. ricinus* larvae that fed on *B. afzelii*-infected mice had a significantly faster larva-to-nymph moulting time compared to larvae that fed on uninfected control mice, but the effect was small (2.4% reduction) and unlikely to be biologically significant. We found no evidence that *B. afzelii* infection or reduction of the larval microbiota influenced the four other life history traits of the immature *I. ricinus* ticks, which included engorged larval weight, unfed nymphal weight, larva-to-nymph moulting success, and immature tick survival. A retrospective power analysis found that our sampling effort had sufficient power (> 80%) to detect small effects (differences of 5% to 10%) of our treatments. Under the environmental conditions of this study, we conclude that *B. afzelii* and the egg surface microbiota had no meaningful effects on tick fitness and hence on the R_0_ of Lyme borreliosis.

## Introduction

The epidemiology of vector-borne diseases is highly sensitive to the biology of the arthropod vector. Theoretical models of the reproduction number (R_0_) of vector-borne diseases show that this quantity depends on several vector behavioural and life history traits, such as the biting rate, survival, and reproduction^1–3^. Infection by vector-borne pathogens can reduce the survival and reproduction of the arthropod vector^4–7^, and thereby reduce the R_0_ of the vector-borne disease. Conversely, vector-borne pathogens can manipulate their arthropod vectors (e.g., increase the biting rate, shift resources from reproduction to survival) to increase their own transmission, and thereby increase the R_0_ of the vector-borne disease^8–11^. Measuring the effects of vector-borne pathogens on the fitness of their arthropod vectors is therefore important for understanding the epidemiology of vector-borne diseases.

Arthropod vectors also form intimate associations with microorganisms that do not cause diseases in vertebrate hosts^12^. These non-pathogenic bacteria are the microbiota of the arthropod vector and they can be inherited from the mother (i.e., vertical transmission) or acquired from the external environment^13^. Some members of the arthropod microbiota are obligate symbionts that have a critical function; for example, providing a vitamin that is missing from a nutrient-poor hematophagous (blood-based) diet^14^. The importance of arthropod-symbiont associations can be demonstrated with dysbiosis treatments (e.g., antibiotics) that decrease both the abundance of a critical symbiont and the fitness of the arthropod vector^15–20^. The arthropod-associated microbiota can also influence vector competence, which is the ability of the arthropod vector to acquire, maintain, and transmit vector-borne pathogens over its lifecycle^21–25^. Thus, arthropod-associated bacteria can influence the R_0_ of vector-borne diseases by their effects on both vector fitness and vector competence for vector-borne pathogens.

Hard ticks of the genus *Ixodes* transmit a diversity of tick-borne pathogens such as the spirochete bacteria belonging to the *Borrelia burgdorferi* sensu lato (sl) genospecies complex, which includes the causative agents of Lyme borreliosis^3,26,27^. Some reviews have suggested that *B. burgdorferi* sl can adaptively manipulate its *Ixodes* tick vector to enhance its own transmission, and hence the R_0_ of Lyme borreliosis^11,28^. *Ixodes* ticks infected with *B. burgdorferi* sl differ from their uninfected counterparts in several phenotypes including questing behaviour, survival, body weight, and energy reserves^29–35^. However, most of these studies are correlational in nature, and there is a lack of controlled infection experiments investigating the effects of *B. burgdorferi* sl on the fitness of their tick vectors. *Ixodes* ticks also contain a diverse microbiota of non-pathogenic bacteria^36,37^, but their effects on tick fitness are even less clear^38,39^.

A basic grasp of the tick life cycle is necessary to understand how *B. burgdorferi* sl and the tick-associated microbiota can influence the fitness of *Ixodes* ticks and hence the R_0_ of Lyme borreliosis^1,3^. *Ixodes* ticks have three motile stages, larva, nymph, and adult female, that must take a blood meal from a vertebrate host to graduate to the next step in the lifecycle. Larvae acquire *B. burgdorferi* sl after engorging on an infected host, and subsequently moult into infected nymphs. The size of the larval blood meal determines the body size and energy reserves of the resultant nymph, which influence nymphal survival and the ability to search for a host the following year^28,34^. The density of infected nymphs (DIN) largely determines the risk of exposure to *B. burgdorferi* sl for vertebrate hosts including humans^40^. Adult female ticks typically feed on medium-sized to large mammals (e.g., deer) that are not competent to host *B. burgdorferi* sl ^41,42^ and their main contribution to the R_0_ of Lyme borreliosis is the production of eggs that hatch into larvae. In summary, the R_0_ of *B. burgdorferi* sl is highly sensitive to the life history traits of immature *Ixodes* ticks, such as larva-to-nymph moulting success, survival of immature ticks, and body weight of immature ticks^1–3^.

In Europe, *B. afzelii* is one of the most common causes of Lyme borreliosis^43^. *B. afzelii* is transmitted by the sheep tick, *Ixodes ricinus*, and its preferred reservoir hosts are small mammals, such as rodents and insectivores^43^. Our research group uses experimental infections with *B. afzelii*, the tick vector *I. ricinus*, and rodent reservoir hosts to ask questions about the ecology of this important tick-borne pathogen^44–50^. The purpose of the present study was to test whether infection with *B. afzelii* and the tick-associated microbiota influence the life history traits of immature *I. ricinus* ticks under standard laboratory conditions.

## Materials and methods

### Background of the study

In a previous study, we investigated whether manipulation of the microbiota of *I. ricinus* larvae would affect their ability to acquire *B. afzelii* during the larval blood meal (i.e., vector competence)^51^. The microbiota of *I. ricinus* larvae was manipulated by surface sterilizing eggs with bleach and ethanol, whereas the control eggs were washed with water. The resultant dysbiosed larvae and control larvae were fed on either *B. afzelii*-infected mice or uninfected control mice and the resultant engorged larvae were allowed to moult into nymphs under laboratory conditions^51^. In addition to the interactions between *B. afzelii* and the tick microbiota presented in^51^, we measured five life history traits (or fitness traits) including (i) engorged larval weight, (ii) unfed nymphal weight, (iii) larva-to-nymph moulting success, (iv) larva-to-nymph moulting time, and (v) immature tick survival. In the present study, we test whether *B. afzelii* infection and manipulation of the larval microbiota influence the fitness of immature *I. ricinus* ticks under laboratory conditions.

### Mice, *Borrelia afzelii*, and *Ixodes ricinus* ticks

We used *Mus musculus* BALB/c mice as the rodent host because we have a history of successful experimental infections with this mouse strain^49,50,52^. During the study, the mice were maintained in individual cages and were given food and water ad libitum. The mice were experimentally infected via tick bite with *B. afzelii* strain NE4049. This strain was originally isolated from an *I. ricinus* tick in Neuchâtel, Switzerland and has multilocus sequence type (MLST) 679, *ospC* major group A10, and strain identification number 1887 in the *Borrelia* MLST database. We chose to work with strain NE4049 because it is highly infectious to both rodents and *I. ricinus* ticks^45,49,50^.

The microbiota of ticks in nature is expected to be more diverse compared to the microbiota of laboratory tick colonies with a long history of domestication^53^. For this reason, we decided to manipulate the microbiota in *I. ricinus* larvae produced by field-collected adult female ticks rather than using larvae from a laboratory colony. The adult female *I. ricinus* ticks used to produce the eggs and larvae for this study were collected from wild roe deer captured in the Sylve d’Argenson forest near Chizé, France. In contrast, to infect mice via tick bite, the ticks must be from a pathogen-free tick population. For this reason, the *I. ricinus* nymphs used to experimentally infect the mice with *B. afzelii* strain NE4049 came from our laboratory colony of *I. ricinus* established in 1978 at the University of Neuchâtel.

All tick stages (adult females, eggs, larvae, and nymphs) were kept in a phytotron whenever they were not being manipulated. The phytotron conditions consisted of a 16-h light phase with a temperature of 25 °C and an 8-h dark phase with a temperature of 18 °C. There was a 1-h phase at dawn and dusk where the temperature was 21.5 °C.

### Approval for the use of experimental animals in the study

*Ixodes ricinus* ticks were collected from roe deer (*Capreolus capreolus*) in strict accordance with the recommendations of the French National Charter on the ethics of animal experimentation and the Directive 2010/63/EU of the European Parliament and of the Council of 22 September 2010 on the protection of animals used for scientific purposes. The “Comité d’Ethique en Expérimentation Animale de l’Université Claude Bernard Lyon 1” approved the animal use protocol (CEEA-55; DR2014-09).

The authors complied with the ARRIVE guidelines. The experimental infection part of the study followed the Swiss legislation on animal experimentation. The commission that is part of the “Service de la Consommation et des Affaires Vétérinaires (SCAV)” of the canton of Vaud, Switzerland evaluated and approved the ethics of this study. The SCAV of the canton of Neuchâtel, Switzerland issued the animal experimentation permits for the study (NE04-2014) and for the maintenance of the *I. ricinus* tick colony on vertebrate hosts at the University of Neuchâtel (NE05-2014).

### Surface sterilization of the egg clutches of field-collected female *I. ricinus* ticks

Surface sterilization of arthropod eggs can be used to manipulate the microbiota of the emerging arthropod larvae^54–58^. These treatments typically bathe the arthropod eggs in sterilizing solutions like 10% bleach or 70% ethanol for a few minutes to kill the bacteria that are colonizing the outer surface of the egg^54–58^. As we were concerned that egg surface sterilization would reduce the hatching success of the *I. ricinus* larvae, we performed a pilot study where the eggs of *I. ricinus* were treated with a variety of surface sterilization treatments that differed with respect to the duration that the eggs were bathed in bleach solution and its concentration (see Section 1 of the electronic supplementary material (ESM) for details). This pilot study found that the egg surface sterilization treatments had no negative effects on the hatching success of the *I. ricinus* larvae, and we therefore chose the harshest treatment for our main study (Section 1 of the ESM).

**Figure 1:**
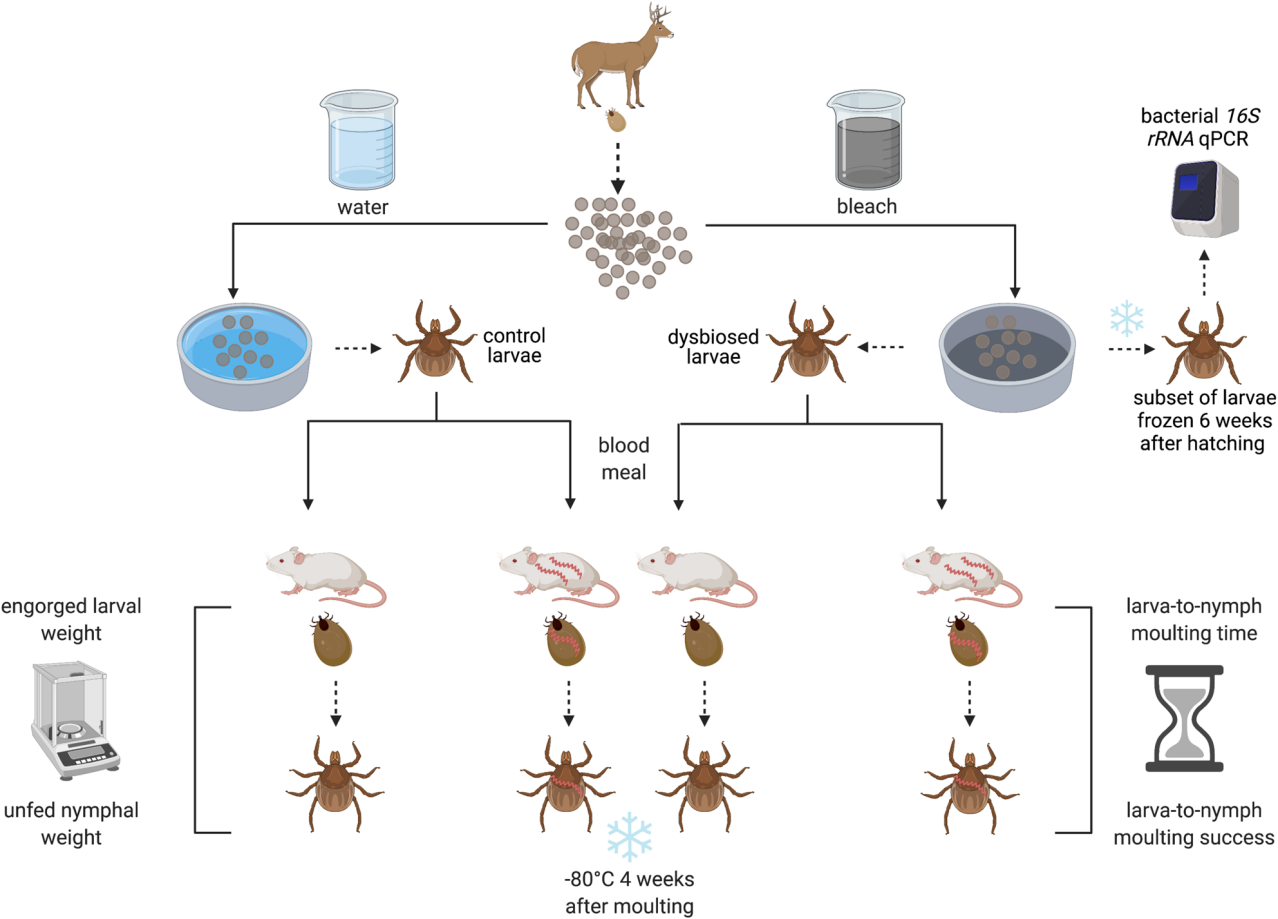
Experimental design of the study. Engorged adult female *I. ricinus* ticks (n = 10) were collected from roe deer captured near Chizé, France and laid their eggs in the laboratory. Each of the 10 egg clutches was split into two batches; one batch of eggs was surface-sterilized (washed with 10% bleach and 70% ethanol) and hatched into dysbiosed larvae, the other batch of eggs received a control treatment (washed with distilled water) and hatched into control larvae. To determine whether egg surface sterilization influenced the larval microbiota, subsets of dysbiosed larvae and control larvae were frozen at 6 weeks after hatching; these larvae were tested for the abundance of their bacterial microbiota using *16S rRNA* qPCR. Larvae for each of the 20 batches (10 tick families × 2 egg surface treatments) were split into two groups of ~ 100 larvae (total of 40 groups) that were fed on either an uninfected control mouse (n = 20) or a *B. afzelii*-infected mouse (n = 20). For each of the 40 mice, up to 60 engorged larvae were placed in individual Eppendorf tubes and left to moult into unfed nymphs. The larva-to-nymph moulting success and survival of these engorged larvae (n = 1739) was monitored over a period of 74 days. For each of the 40 mice, up to 20 engorged larvae were randomly selected to be weighed as engorged larvae and again as unfed nymphs at 4 weeks after the larva-to-nymph moult. A subset of ~ 300 nymphs was frozen at – 80 °C and tested for *B. afzelii* infection using qPCR. This figure was created using BioRender.com.

Ten engorged adult female *I. ricinus* ticks were collected from wild roe deer captured in the Sylve d’Argenson forest near Chizé, France. These 10 engorged adult female ticks were transported to the University of Neuchâtel where they laid their eggs in the phytotron (Fig. 1). Four weeks after egg laying, each of the 10 clutches of *I. ricinus* eggs was split into two batches; one batch was exposed to the egg surface sterilization treatment, whereas the other batch was exposed to the egg surface control treatment. In the egg surface sterilization treatment, the eggs were bathed in a 10% bleach solution for 5 min, a 70% ethanol solution for 3 min, before being gently rinsed with distilled water for 3 min; in the control treatment, the 10% bleach and 70% ethanol solutions were replaced with distilled water (see Section 2 of the ESM for details). After the egg surface treatment, the eggs were placed in plastic petri dishes sealed with parafilm and left to hatch into *I. ricinus* larvae in the phytotron. Thus, for each of the 10 tick families, we obtained one batch of dysbiosed larvae that hatched from the surface sterilized eggs (bathed in bleach and ethanol) and one batch of control larvae that hatched from the control eggs (bathed in distilled water) for a total of 20 batches of larvae. We did not observe any obvious differences in egg hatching or larval viability between the egg surface sterilization group versus the control group. To determine whether the egg surface sterilization treatment had reduced the microbiota in the *I. ricinus* larvae, a subset of ~ 200 larvae was frozen at 6 weeks after hatching for each of the 20 batches of larvae. The remaining dysbiosed larvae and control larvae were fed on mice (*B. afzelii*-infected or uninfected) and were subsequently measured with respect to their life history traits, as described below.

### Experimental infection of mice with *B. afzelii* via nymphal tick bite

For the main experiment, 40 female, 5-week-old, specific pathogen-free BALB/c mice were randomly assigned to either the control group or the infection group (Fig. 1). To simulate the natural life cycle of *B. afzelii*, the mice were experimentally infected via nymphal tick bite rather than needle inoculation. We used the University of Neuchâtel laboratory colony of *I. ricinus* to create uninfected nymphs and nymphs infected with *B. afzelii* strain NE4049 (see Section 3 of the ESM for details), as we have done previously^44–46,48,50^. Each mouse in the control group (n = 20) was infested with 5 uninfected *I. ricinus* nymphs, whereas each mouse in the infected group (n = 20) was infested with 5 nymphs putatively infected with *B. afzelii* strain NE4049. In this way, each of the 40 mice had a similar immune experience with *I. ricinus* nymphs. Five weeks after the nymphal challenge, an ear tissue biopsy and a blood sample were taken from each of the 40 mice to determine their infection status. The ear tissue biopsy was tested for the presence of *B. afzelii* using qPCR. The blood sample was tested for the presence of *B. afzelii*-specific antibodies using a commercial ELISA. These tests confirmed that the 20 mice in the infected group were infected with *B. afzelii*, whereas the 20 mice in the control group were uninfected. These 40 mice were used to feed the remaining dysbiosed larvae and control larvae that had hatched from the surface-sterilized eggs and the control eggs, respectively.

### Feeding dysbiosed and control *I. ricinus* larvae on *B. afzelii*-infected and uninfected control mice

As mentioned previously, for each of the 10 tick families, we obtained dysbiosed larvae that hatched from surface-sterilized eggs and control larvae that hatched from control eggs (10 families × 2 egg surface treatments = 20 batches of larvae). The larvae for each of the 20 batches were split into two groups of ~ 100 larvae (i.e., total of 40 groups of larvae). One group was fed on an uninfected control mouse, whereas the other group was fed on a *B. afzelii*-infected mouse (Fig. 1). We used a paint brush to pick up ~ 100 larvae (the number of larvae were visually estimated and not counted exactly) and to brush them onto each mouse. During the larval infestation, mice were anaesthetized with a mixture of xylazine, ketamine, and PBS (1:2:9; 5 μl per gram of mouse), as we have done previously^44–46,48,50^. The *I. ricinus* larvae blood fed for 3 days on the mice before dropping off. To facilitate the collection of the engorged larvae, we removed the micro-isolator top from the mouse cage and placed the mouse cage in a larger rat cage that contained a shallow moat of water. This set-up allows the engorged larvae to climb out of the mouse cage, through the wire metal top, and fall into the water moat, where they are easily collected with a paint brush. We collected a maximum of 60 engorged larvae for each mouse. Each engorged larva was placed in an individual Eppendorf tube and was left to moult into a nymph. The Eppendorf tubes contained a piece of moistened paper towel to ensure high relative humidity and were organized in cryoboxes that were stored in the phytotron. Four weeks after the larva-to-nymph moult, 7 to 8 nymphs were randomly selected for each mouse and were frozen at – 80 °C. In summary, we froze ~ 300 nymphs (10 families × 2 egg surface treatments × 2 mouse infection statuses ×  (7–8) nymphs/mouse) for future molecular analyses of *B. afzelii* infection.

### Life history traits of immature *I. ricinus* ticks

One of the 40 mice died during the larval infestation. We collected a total of 1739 engorged *I. ricinus* larvae from the remaining 39 mice (mean = 44.6; range = 5 to 60; units are engorged larvae per mouse). These 1739 engorged larvae belonged to 4 different groups: (i) control larvae that fed on uninfected mice (n = 419 larvae), (ii) control larvae that fed on *B. afzelii*-infected mice (n = 429 larvae), (iii) dysbiosed larvae that fed on uninfected mice (n = 449 larvae), and (iv) dysbiosed larvae that fed on *B. afzelii*-infected mice (n = 442 larvae). These engorged larvae were kept in individual Eppendorf tubes that were labelled with a unique tick ID so that we could follow their moulting status and survival status over time.

To determine the larva-to-nymph moulting success and immature tick survival, the moulting status and survival status of the engorged larvae (n = 1739) were both checked at 74 days after the larval infestation. The larva-to-nymph moulting time was calculated as the time interval between the date of the larval infestation and the date that the tick was first observed to be a nymph. We chose the date of the larval infestation rather than the date of larval drop off because we knew the exact date of the former but not the latter. We checked the moulting status of the ticks every 2 or 3 days over a period of 54 days after the larval infestation to get a precise estimate of the larva-to-nymph moulting date for 1529 of the 1739 ticks. We did not check the ticks over the next 20 days, and as we did not get a precise estimate of the larva-to-nymph moulting date for the remaining 210 ticks (1739–1529), we excluded them from the analysis of the larva-to-nymph moulting time.

For engorged larval weight and unfed nymphal weight, we weighed a smaller subset of ticks. For each mouse, we randomly selected a maximum of 20 engorged larvae and weighed them within 2 days of their collection (n = 742 engorged larvae). Four weeks after the larva-to-nymph moult, we weighed the same group of nymphs that had been previously weighed as engorged larvae (n = 742–89 = 653 unfed nymphs because 89 engorged larvae did not moult into nymphs). To increase the sample size, we weighed an additional 414 unfed nymphs (~ 10 nymphs for each of the 40 mice) so that the total sample size was 1067 nymphs (653+414 = 1067). The engorged larvae and the unfed nymphs were weighed to a precision of 0.1 μg using an Ultra Microbalance (UMT 5 Comparator, Mettler Toledo, Greifensee, Switzerland).

In summary, we collected five life history traits for these immature *I. ricinus* ticks: (i) engorged larval weight within 2 days of drop-off (n = 742), (ii) unfed nymphal weight at 4 weeks after the larva-to-nymph moult (n = 1067), (iii) larva-to-nymph moulting success over 74 days following the start of the larval blood meal (n = 1739), (iv) larva-to-nymph moulting time over 54 days following the start of the larval blood meal (n = 1529), and (v) immature tick survival over 74 days following the start of the larval blood meal (n = 1739). The numbers of engorged larvae that were placed in individual Eppendorf tubes (maximum of 60) were recorded for each of the 39 mice, and this variable is an index of the larval infestation success.

### Molecular methods for unfed dysbiosed and control *I. ricinus* larvae

As mentioned previously, for each of the 20 batches of larvae (10 families × 2 egg surface treatments = 20 batches), we had frozen a subset of ~ 200 larvae at 6 weeks after hatching for molecular analysis. DNA extraction of these 20 subsets of frozen larvae was done in 40 pools (~ 100 larvae per pool) using a DNeasy Blood & Tissue spin-column kit (QIAGEN) and following the manufacturer’s instructions. The DNA of each pool was eluted into 100 μl of distilled water, and the DNA concentration was measured using a Nanodrop. For qPCR, the DNA concentration of each pool was adjusted to 5 ng/μl. Two qPCR assays were performed independently for each pool of larvae: tick *calreticulin* and bacterial *16S rRNA*. Each qPCR assay contained 3 μl of template for a total of 15 ng of DNA. The molecular methods are described in detail in Section 4 of the ESM.

### Molecular methods for unfed *I. ricinus* nymphs

As mentioned previously, for each of the 40 mice, a sample of 7 to 8 unfed nymphs (that had fed as larvae on the mice) had been randomly selected and frozen for molecular analysis at 4 weeks after the larva-to-nymph moult. Nymphs were crushed using the TissueLyser II (QIAGEN) by shaking them with a stainless steel bead (1.4 mm in diameter) at a frequency of 30 Hz for 1 min. Total DNA was extracted for each nymph using the DNeasy Blood & Tissue 96-well extraction kit (QIAGEN) and following the manufacturer’s instructions. The DNA of each nymph was eluted into 65 μl of distilled water, and the DNA concentration was measured for each of the ~ 300 nymphs using a Nanodrop. For qPCR, the DNA concentration of each nymph was adjusted to 5 ng/μl. Three qPCR assays were performed independently for each nymph: tick *calreticulin*, bacterial *16S rRNA*, and *B. burgdorferi* sl *flagellin*. Each qPCR assay contained 3 μl of template for a total of 15 ng of DNA. The molecular methods are described in detail in Section 4 of the ESM.

### ELISA to determine whether mice developed IgG antibodies against *B. afzelii*

We used the SERION ELISA classic *B. burgdorferi* sl IgG/IgM immunoassay to detect the presence of IgG antibodies against *B. afzelii*, as we have described previously^50^. The strength of the IgG antibody response against *B. afzelii* was measured in absorbance units (AU). The ELISA is described in detail in Section 4 of the ESM.

### Statistical methods

Statistical analyses were performed using RStudio Version 1.2.5042^59^. The Linear Models (LMs) and the Generalized Linear Models (GLMs) were created using the *lm()* and *glm()* functions in the base package ^59^. The Linear Mixed effects Models (LMMs) and the Generalized Linear Mixed effects Models (GLMMs) were created using the *lmer()* and *glmer()* functions in the lme4 package^60^. The correlation matrix and the p-values were calculated using the *rcorr()* function in the Hmisc package^61^. The graph of the correlation matrix of the life history traits of the immature *I. ricinus* ticks was created using the *corrplot()* function in the corrplot package^62^. Effects with p-values < 0.050 were considered statistically significant. After Bonferroni correction for five life history traits, p-values < 0.010 were considered statistically significant.

### Effect of egg surface sterilization treatment on the microbiota of *I. ricinus* larvae

For each of the 40 pools of *I. ricinus* larvae (~ 100 larvae per pool), we divided the bacterial *16S rRNA* gene copy number by the tick *calreticulin* gene copy number. These *16S rRNA* to *calreticulin* ratios were then log10-transformed to improve the normality of the data. To test whether the egg surface sterilization treatment reduced the microbiota of the resultant *I. ricinus* larvae, the log10-transformed *16S rRNA* to *calreticulin* ratios were analysed using LMMs. Egg surface treatment (2 levels: surface sterilization and control) was modelled as a fixed factor. The larvae that are the offspring from the 10 field-collected adult females represent 10 different tick families, and this factor was modelled as a random effect to account for the non-independence among relatives.

### Effect of *B. afzelii* infection and egg surface sterilization treatment on the life history traits of immature *I. ricinus* ticks

The response variables were the five life history traits of the immature *I. ricinus* ticks: (i) engorged larval weight within 2 days of drop-off (units are μg), (ii) unfed nymphal weight at 4 weeks after the larva-to-nymph moult (units are μg), (iii) larva-to-nymph moulting success over 74 days following the start of the larval blood meal (units are %), (iv) larva-to-nymph moulting time over 54 days following the start of the larval blood meal (units are days), and (v) immature tick survival over 74 days following the start of the larval blood meal (units are %). We used mixed effects models to analyse these response variables to account for the non-independence of ticks that are from the same tick family and that fed on the same mouse. The log10-transformed engorged larval weight, log10-transformed unfed nymphal weight, and larva-to-nymph moulting time were treated as normally distributed response variables that were analysed using LMMs. The larva-to-nymph moulting success and immature tick survival are binomial response variables that were analysed using GLMMs. Egg surface treatment (2 levels: surface sterilization and control) and mouse infection status (2 levels: infected and control) were modelled as fixed effects. Mouse ID and tick family were modelled as random effects, with mouse ID nested in tick family. If the interaction between egg surface treatment and mouse infection status was not significant, the model was re-run without the interaction.

### Effect of tick family and mouse ID on the life history traits of immature *I. ricinus* ticks

To test whether there were significant differences among the 10 tick families, the five life history traits were modelled using LMMs with normal errors or GLMMs with binomial errors with tick family as a fixed effect and mouse ID as a random effect. To test whether there were significant differences among the 40 mice, the 5 life history traits were modelled as LMs with normal errors or GLMs with binomial errors with mouse ID as a fixed effect. For engorged larval weight and unfed nymphal weight, we estimated the variance components due to tick family, mouse ID, and the individual tick by using LMMs with the random effect of mouse ID nested in the random effect of tick family.

### Correlations between the life history traits of immature *I. ricinus* ticks

For this correlation analysis, there were six variables: the number of engorged larvae that were recovered from each mouse (maximum of 60) and the five previously mentioned life history traits of the immature *I. ricinus* ticks. For each of these six variables, the mean was calculated for each of the 40 mice. The pairwise correlations were calculated for each of the 15 pairs of variables across the 40 mice.

## Results

### Effect of egg surface sterilization on the microbiota of *I. ricinus* larvae

**Figure 2:**
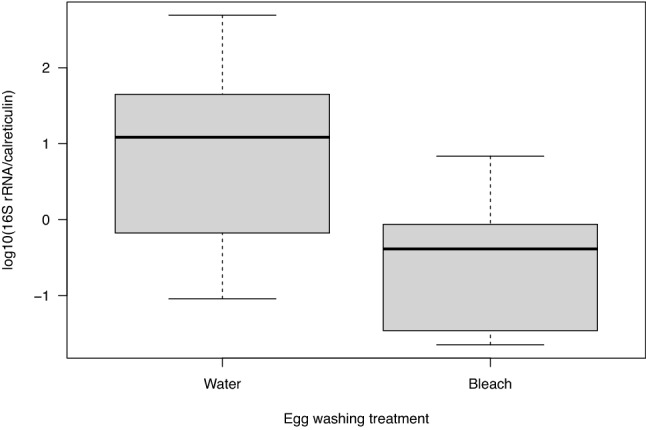
The egg surface sterilization treatment reduced the bacterial microbiota of the *I. ricinus* larvae. The labels ‘Water’ and ‘Bleach’ (on the X-axis) refer to the control and surface sterilization treatments that were applied to the eggs that had been laid by the 10 engorged adult female *I. ricinus* ticks. For each of the 20 batches of larvae (10 tick families × 2 egg surface treatments), we froze subsets of  ~ 200 larvae at 6 weeks after hatching and extracted the DNA in pools of ~ 100 larvae (total of 40 pools). We used qPCR to determine the ratio of the bacterial *16S rRNA* gene copy number to the tick *calreticulin* gene copy number, which measures the abundance of the tick-associated bacterial microbiota. This ratio was 28 times higher for the control larvae that hatched from the control eggs (‘Water’ treatment) compared to the dysbiosed larvae that hatched from the surface-sterilized eggs (‘Bleach’ treatment), and this difference was highly significant (p = 0.0000005). The ratios of the bacterial *16S rRNA* to tick *calreticulin* gene copy numbers were log10-transformed to improve normality and the appearance of the graph. The boxplots show the median (black line), 25th and 75th percentiles (edges of the box), and minimum and maximum values (whiskers).

The *I. ricinus* larvae that hatched from the eggs that were surface sterilized with bleach and ethanol had a significantly lower bacterial abundance than the control larvae that hatched from the control eggs washed with water (Fig. 2; χ^2^ = 25.359, df = 1, p = 0.0000005). The log10(*16S rRNA*/ *calreticulin*) ratio of the larvae in the control group (mean ratio = 6.61, 95% CI = 1.78–24.61) was 28 times higher compared to the egg surface sterilization group (mean ratio = 0.24, 95% CI = 0.06–0.88). The egg surface sterilization treatment with bleach and ethanol was therefore highly effective at reducing the microbiota in the *I. ricinus* larvae that hatched from these surface sterilized eggs (Fig. 2).

### *Borrelia afzelii* induced a strong IgG antibody response in the infected mice

**Figure 3:**
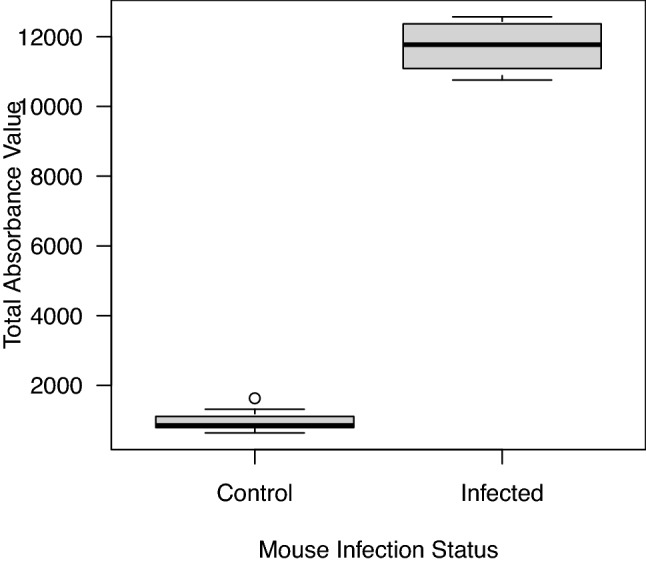
* Borrelia afzelii* induces a strong IgG antibody response in the infected mice compared to the uninfected control mice. The total absorbance values were obtained from a commercially available ELISA that measured the strength of the IgG antibody response against *B. afzelii*. The mean IgG antibody response against* B. afzelii* of the infected mice (11,749 absorbance units (AU)) was 12.9 times higher compared to the control mice (912 AU), and this difference was highly significant (p < 0.000001). The boxplots show the median (black line), 25th and 75th percentiles (edges of the box), minimum and maximum values (whiskers), and outliers (open circles).

One of the control mice died during the study, so that the final sample sizes were 19 uninfected control mice and 20 *B. afzelii*-infected mice. The strength of the IgG antibody response to *B. afzelii* in the mice was measured in absorbance units (AU). The ability of the ELISA to detect *B. afzelii* infection in the mice was unambiguous (Fig. 3) because there was no overlap in absorbance between the control group (range = 637 to 1692 AU) versus the infected group (range = 10,756 to 12,572 AU). According to the ELISA, all the 20 mice challenged with infected nymphs became infected with *B. afzelii* strain NE4049 and all the 19 mice challenged with uninfected nymphs remained uninfected (Fig. 3). The mean IgG antibody response against *B. afzelii* of the infected mice (11,749 AU) was significantly higher (12.9×) compared to the control mice (912 AU; independent samples t-test: t = 43.88, df = 37, p < 0.000001).

### Prevalence of *B. afzelii* infection in unfed *I. ricinus* nymphs

The dysbiosed and control larvae that had fed on the *B. afzelii*-infected mice or the uninfected control mice had been left to moult into unfed nymphs. We extracted DNA from ~ 300 unfed nymphs, and a subset of 289 unfed nymphs with sufficiently high DNA concentrations was tested for *B. afzelii* via qPCR. Of the 289 nymphs, 106 nymphs had fed as larvae on uninfected control mice and 183 nymphs had fed as larvae on infected mice. The prevalence of *B. afzelii* infection in the nymphs that had fed as larvae on the infected mice (71.0% = 130/183) was significantly higher (25.1x) compared to the nymphs that had fed as larvae on the uninfected control mice (2.8% = 3/106; proportion test: χ^2^ = 122.97, df = 1, p < 0.000001). The 3 nymphs that had fed as larvae on the uninfected control mice, but that tested positive for *B. afzelii* on the qPCR are false positives (these 3 nymphs had high Cq values of 39.62, 39.74, and 40.22; see Section 5 of the ESM). Data on the variation in host-to-tick transmission of *B. afzelii* among the 20 infected mice is given in Section 6 of the ESM.

### Relationship between engorged larval weight and unfed nymphal weight

**Figure 4:**
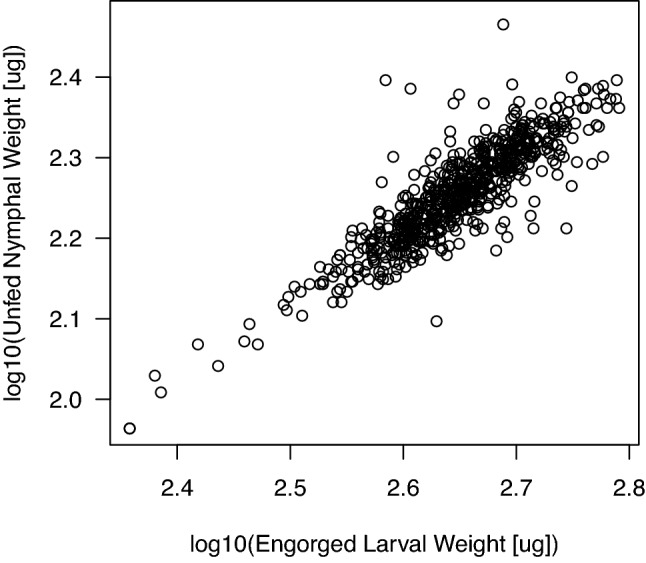
The weight of the unfed *I. ricinus* nymphs depends on the weight of the engorged larvae. The engorged larval weights and unfed nymphal weights were measured ~ 10 weeks apart for a sample of 653 immature *I. ricinus* ticks. The positive relationship between the log10-transformed unfed nymphal weights (μg) and the log10-transformed engorged larval weights (μg) is highly significant (r = 0.854, t = 41.859, df = 652, p < 0.000001).

The engorged larval weights and unfed nymphal weights were both measured for a sample of 653 *I. ricinus* ticks. The log10-transformed engorged larval weight was a highly significant predictor of the log10-transformed unfed nymphal weight, and the former explained 72.7% of the variation in the latter (Fig. 4; regression: F_1, 651_ = 1738, p < 0.000001; correlation: r = 0.854, t = 41.859, df = 652, p < 0.000001). Given that the engorged larvae and unfed nymphs were weighed independently and more than 10 weeks apart, the strong relationship between these two variables indicates that our weight measurements of the immature ticks are highly reliable. For this paired sample of 653 immature ticks, the mean weight of the engorged larvae (438.7 μg) was 2.4× greater than the resultant unfed nymphs (180.8 μg), and this difference was highly significant (paired t-test: t = − 229.53, df = 652, p < 0.000001). These results show that the engorged larvae lost 58.8% of their weight during the larva-to-nymph moulting process (i.e., due to water loss, digestion of blood, and excretion of faecal material). We found no evidence that *B. afzelii* infection in the mouse influenced the efficiency with which the engorged larval weight was converted into unfed nymphal weight (see Section 7 of the ESM).

A previous study found that *I. scapularis* larvae that took larger blood meals had a higher probability to acquire *B. burgdorferi* sensu stricto (ss)^63^. To test this hypothesis, we analyzed the probability that the *I. ricinus larvae * acquired *B. afzelii* as a function of the size of the larval blood meal for the subset of larvae that fed on the 20 infected mice. We found no evidence that the size of the larval blood meal influenced the probability that the larvae acquired *B. afzelii* (see Section 8 of the ESM).

### Larva-to-nymph survival, moulting success, and moulting time

Of the 1739 engorged larvae that we monitored, the mean larva-to-nymph moulting success (monitored over an interval of 74 days) was 91.9% (1598/1739), and the mean immature tick survival (monitored over an interval of 74 days) was 84.5% (1470/1739). Of the 141 larvae that did not moult, 134 died before reaching the moult, and the other 7 had not moulted after 74 days. Of the 1739 engorged larvae, the moulting time (days) was recorded for 1529 individuals (monitored over an interval of 54 days). The median larva-to-nymph moulting time was 40 days (range = 38 to 54 days). The values of these life history traits are defined for this population of *I. ricinus* from Chizé, France under our laboratory conditions (25 °C, high humidity).

### Effect of *B. afzelii *infection and egg surface sterilization on the life history traits of immature *I. ricinus* ticks

**Figure 5:**
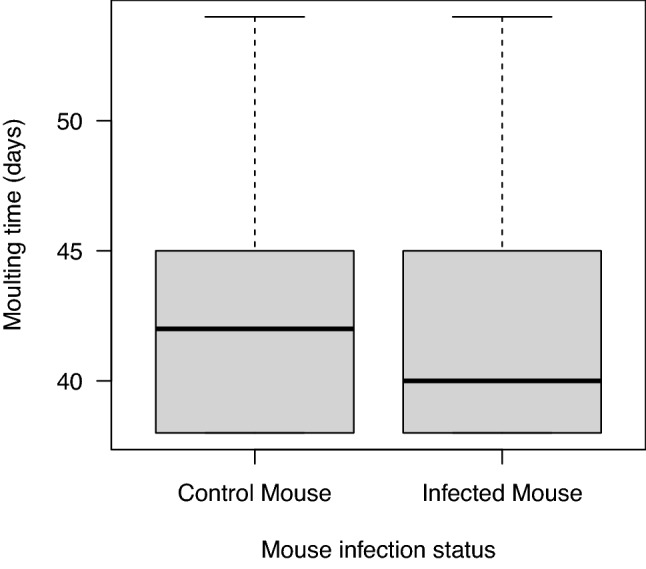
*Ixodes rinicus* larvae that feed on *B. afzelii*-infected mice have a faster larva-to-nymph moulting time compared to larvae that feed on uninfected control mice. The mean larva-to-nymph moulting time (days) for the larvae that fed on the *B. afzelii*-infected mice (mean = 41.3 days; median = 40 days; n = 776 ticks) was 1.0 day faster compared to the larvae that fed on the uninfected control mice (mean = 42.3 days; median = 42 days; n = 753 ticks), and this difference was highly significant (p < 0.0001). The larva-to-nymph moulting time was monitored over a period of 54 days after the start of the larval blood meal. The boxplots show the median (black line), 25th and 75th percentiles (edges of the box), and minimum and maximum values (whiskers).

We found no significant effects of mouse infection status, egg surface treatment, or their interaction on the engorged larval weights or unfed nymphal weights (Table 1; see Section 9 of the ESM). The results remained the same when the explanatory variable of mouse infection status was replaced with tick infection status (see Section 10 of the ESM). No significant effects of mouse infection status, egg surface treatment, or their interaction were found on larva-to-nymph moulting success, larva-to-nymph moulting time, or immature tick survival (Table 1); the only exception was the effect of mouse infection status on the larva-to-nymph moulting time (Fig. 5; Table 1; χ^2^ = 16.510, df = 1, p < 0.0001). The mean larva-to-nymph moulting time for the ticks that fed on the infected mice (41.4 days) was 1.0 day faster (2.4% faster) than the ticks that fed on the uninfected control mice (42.4 days). The effect of *B. afzelii* infection remained significant after using a non-parametric independent two-samples Wilcoxon test (W = 359,542, p < 0.00001; see Section 11 of the ESM) and after Bonferroni correction for multiple comparisons (i.e., p = 0.050/5 = 0.010).

**Table 1:** Statistical analyses of the five life history traits of the immature *I. ricinus* ticks.

Life history trait	Explanatory variable	df	χ^2^	p
Engorged larval weight	MIS: ESS interaction	1	0.414	0.520
Engorged larval weight	MIS	1	0.135	0.714
Engorged larval weight	ESS	1	0.522	0.470
Unfed nymphal weight	MIS: ESS interaction	1	1.131	0.288
Unfed nymphal weight	MIS	1	0.195	0.659
Unfed nymphal weight	ESS	1	0.249	0.618
Moulting success	MIS: ESS interaction	1	0.081	0.775
Moulting success	MIS	1	0.288	0.592
Moulting success	ESS	1	0.037	0.847
Moulting time	MIS: ESS interaction	1	0.648	0.421
Moulting time	MIS	1	16.510	< 0.0001
Moulting time	ESS	1	0.071	0.790
Survival	MIS: ESS interaction	1	0.176	0.675
Survival	MIS	1	0.630	0.427
Survival	ESS	1	0.225	0.635

The five life history traits include engorged larval weight, unfed nymphal weight, larva-to-nymph moulting success, larva-to-nymph moulting time, and immature tick survival. LMMs and GLMMs were used to test the effects of mouse infection status (MIS), egg surface sterilization (ESS), and their interaction (MIS: ESS) on the five life history traits. Shown are the results from the type II log-likelihood ratio test to determine the statistical significance of the explanatory variables. The row headers refer to the life history trait, the explanatory variable, the degrees of freedom (df), the Chi-square statistic (χ^2^), and the p-value (p).

### Effects of tick family and mouse ID on the life history traits of immature *I. ricinus* ticks

We found significant effects of tick family and mouse ID on all five life history traits; the only exception was the effect of tick family on larva-to-nymph moulting success (see Section 12 of the ESM for details). For the engorged larval weight (log10-transformed), variance component analysis found that tick family, mouse ID, and the individual tick accounted for 8.0%, 5.8%, and 86.3% of the variance, respectively. Similarly, for the unfed nymphal weight (log10-transformed), variance component analysis found that tick family, mouse ID, and the individual tick accounted for 7.9%, 4.0% and 88.1% of the variance, respectively. Thus, most of the variance in immature tick weight occurred at the level of the individual ticks and was unexplained.

### Correlations between the life history traits of immature *I. ricinus* ticks

**Figure 6:**
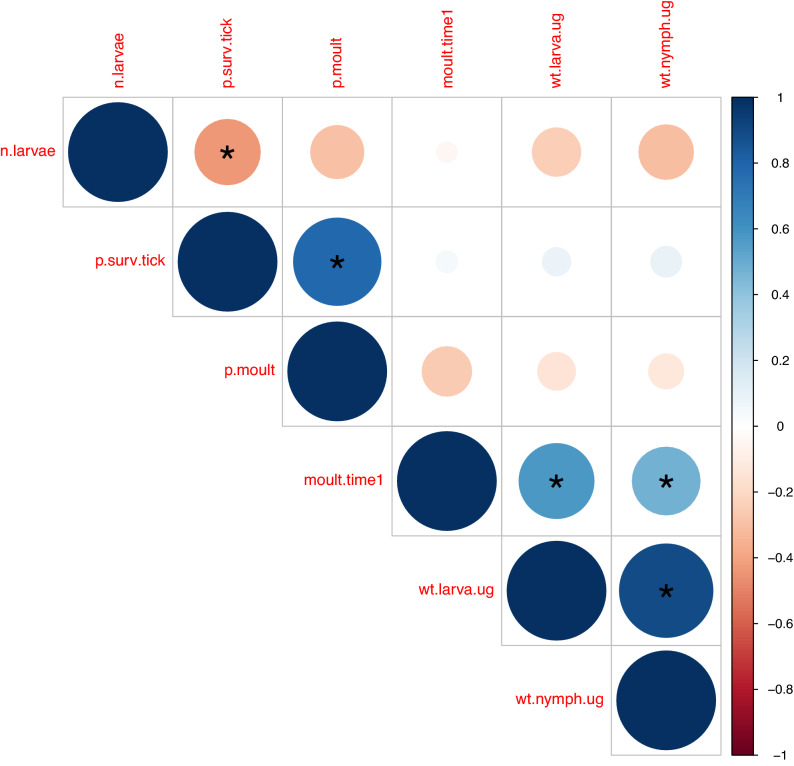
Correlations between the life history traits of the immature *I. ricinus* ticks. The 6 variables shown include (i) number of engorged larvae that were recovered from each mouse (n.larvae), (ii) immature tick survival (p.surv.tick), (iii) larva-to-nymph moulting success (p.moult), (iv) larva-to-nymph moulting time (moult.time1), (v) weight of engorged larvae (wt.larva.ug), and (vi) weight of unfed nymphs (wt.nymph.ug). The correlations were based on the means of the life history traits of the immature ticks for each of the 39 mice. Positive correlations are shown in blue and negative correlations are shown in red. The size of the circle indicates the magnitude of the Pearson correlation coefficient. Statistically significant correlations are indicated with an asterisk.

There were five significant correlations between the six variables, which included the number of engorged larvae that were recovered (maximum of 60) from each mouse and the five life history traits of the immature *I. ricinus* ticks (Fig. 6). The pairwise correlations were calculated using the means of the ticks for each of the 39 mice, and the sample size is therefore 39 for each pairwise correlation. There was a negative correlation between the number of engorged larvae collected and survival of immature ticks (r = −0.438, p = 0.005). The remaining four pairwise correlations were positive: survival of immature ticks and larva-to-nymph moulting success (r = 0.782, p < 0.0001), larva-to-nymph moulting time and engorged larval weight (r = 0.575, p = 0.0002), larva-to-nymph moulting time and unfed nymphal weight (r = 0.471, p = 0.0025), and engorged larval weight and unfed nymphal weight (r = 0.895, p < 0.0001; see also Fig. 4).

### Power analysis to determine the minimal detectable effect size

For each of the five life history traits of the immature *I. ricinus* ticks, we conducted a retrospective power analysis to determine the minimal effect size (of *B. afzelii* infection or egg surface sterilization) that our sampling effort could have detected (see Section 13 of the ESM). This power analysis showed that our sampling effort had a power > 80% to detect a treatment effect (of *B. afzelii* infection or egg surface sterilization) that either increased or decreased the tick phenotype by 10% (relative to the control) for each of the five life history traits. For three life history traits (moulting time, engorged larval weight, and unfed nymphal weight) our sampling effort had a power > 80% to detect a treatment effect (of *B. afzelii* infection or egg surface sterilization) that either increased or decreased the tick phenotype by ~ 5% (relative to the control). In summary, our sampling effort was more than sufficient to detect small positive or negative effects of *B. afzelii* infection or egg surface sterilization on each of the five life history traits.

## Discussion

### Effects of *B. afzelii* on the life history traits of *I. ricinus*

Our study found that the Lyme borreliosis pathogen *B. afzelii* reduced the larva-to-nymph moulting time of its principal tick vector *I. ricinus* by 2.4% (i.e., 1.0 day) under laboratory conditions. In contrast, we found no evidence that infection with *B. afzelii* influenced the other four life history traits of immature *I. ricinus* ticks under laboratory conditions. Similarly, we found no evidence that reduction of the larval microbiota via egg surface sterilization influenced these same life history traits. The five life history traits investigated in this study describe the transition of engorged larvae to unfed nymphs over the first 2.5 months of the tick life cycle, and they are expected to influence the population ecology of *I. ricinus* and hence the R_0_ of *B. afzelii*^1–3^. The sample size differed among the five life history traits and ranged from 742 to 1739 immature *I. ricinus* ticks that had fed as larvae on 40 different mice. Our power analyses demonstrated that our sampling effort was more than sufficient to detect small positive or negative effects (changes of 5% to 10%) of either *B. afzelii* infection or the egg surface sterilization treatment on each of the five life history traits. Thus, under the laboratory conditions used in this study, neither *B. afzelii* infection nor reduction of the larval microbiota (via egg surface sterilization) had major effects (i.e., > 10% difference) on the fitness of immature *I. ricinus* ticks.

### Importance of controlled experimental infections

Our study shows the importance of using controlled experiments to test whether infection with tick-borne pathogens influences the life history traits of their tick vectors. Several studies that reported effects of *B. burgdorferi* sl on *Ixodes* phenotype (e.g., behaviour, body size, fat content, survival) used ticks that had been naturally infected in the field^28,30–34^. This approach cannot control for other confounding factors that influence tick phenotype, such as tick age, vertebrate host species that provided the larval blood meal, quality of the host blood meal, presence of other microbes, etc. For example, vertebrate host species differ in their ability to transmit *B. burgdorferi* ss to feeding *I. scapularis* larvae ^64–67^, and in blood meal quality, which influences variation in *I. scapularis* larva-to-nymph moulting success^68^. Together, these two phenomena can produce significant associations between tick infection status and tick life history traits, which are then misinterpreted as demonstrating that the former causes variation in the latter.

### Effect of *B. afzelii* on larva-to-nymph moulting time of *I. ricinus*

Our study found that *B. afzelii* infection in the mouse reduced the larva-to-nymph moulting time of the engorged *I. ricinus* larvae by 2.4% under laboratory conditions. Although the effect size was small (i.e., 1.0 day), it was highly significant (p < 0.0001). It seems unlikely that a 1-day difference in the larva-to-nymph moulting time could have a meaningful effect on the fitness of *B. afzelii*; nevertheless, we speculate as follows. Faster larva-to-nymph development enhances the fitness of both *I. ricinus* and *B. afzelii*^1–3^. In nature, larvae acquire their blood meal during the summer and then moult into unfed nymphs; most of these nymphs overwinter and quest the following spring, but some nymphs will quest later that same fall^69–72^. The decision of whether to quest that same fall or the following spring depends on the timing of the larval blood meal and the speed of larva-to-nymph development^69–72^. If *B. afzelii* can speed up larva-to-nymph development so that nymphs can quest that same fall rather than the following spring, it would increase the R_0_ of *B. afzelii* and Lyme borreliosis^1–3^. Future studies should investigate whether *B. afzelii* speeds up the larva-to-nymph moulting time under ecologically relevant conditions.

### Effect of *B. afzelii* on moulting success and survival of *I. ricinus*

We found no effect of *B. afzelii* infection on larvae-to-nymph moulting success or immature tick survival. The engorged larvae were kept under favourable environmental conditions in the phytotron (25 °C and high humidity), which may be a limitation of our study if the effects of *B. afzelii* infection on tick fitness are larger under harsher environmental conditions. Studies using field-collected *I. ricinus* ticks found that nymphs infected with *B. burgdorferi* sl have higher survival compared to uninfected nymphs under stressful conditions of humidity and temperature^32^, and that *B. burgdorferi* sl-infected nymphs survive better under cold temperatures and high-frequency temperature variations^33^. A study on another tick-borne bacterium, *Anaplasma phagocytophilum*, found that infection reduced the moulting success and survival of immature *I. scapularis* ticks under laboratory conditions^5^. In contrast, another study found that *A. phagocytophilum* increased the expression of a tick antifreeze glycoprotein, which enhanced survival of engorged *I. scapularis* larvae under cold temperature conditions^73^. These contradictory results demonstrate that the environmental conditions may influence the effect that tick-borne pathogens have on the life history traits of their tick vectors.

### Effects of *B. afzelii* on body size of *I. ricinus*

We found no effect of *B. afzelii* infection on the body size of immature *I. ricinus* ticks. Our results contradict previous studies that found that immature *Ixodes* ticks infected with *B. burgdorferi* sl were larger than uninfected ticks^34,35,63^. A study on field-collected *I. ricinus* ticks found that infected nymphs had a larger body size and higher energy reserves compared to uninfected nymphs^34^. Similarly, an experimental study found that *I. ricinus* nymphs that had acquired *B. afzelii* during the larval blood meal from infected bank voles were heavier than the corresponding uninfected nymphs^35^. This result led the authors to suggest that *B. afzelii* manipulates the larvae to take larger blood meals^35^. Another experimental study with *I. scapularis*, *B. burgdorferi* ss, and mice found a positive association between the size of the larval blood meal and the probability that the tick tested positive for *B. burgdorferi* ss^63^. The authors proposed two hypotheses: *B. burgdorferi* ss affects the larval feeding process or larvae that take larger blood meals are more likely to acquire *B. burgdorferi* ss^63^. In the first hypothesis, the pathogen causes the difference in the size of the vector blood meal between infected and uninfected vectors, whereas in the second hypothesis, the variation in the size of the vector blood meal causes the variation in vector infection status. Our study found no evidence for either hypothesis.

### Scope of selection for manipulation of arthropod vectors by vector-borne pathogens

Hematophagous (blood-feeding) insects and ticks differ dramatically in their biology (e.g., mobility, host seeking behaviour, number and duration of blood meals, etc.) and the scope of any potential manipulation by a vector-borne pathogen is therefore expected to differ between the two types of vectors^1^. In mosquito-malaria systems, the malaria parasite manipulates the mosquito vector to increase its biting rate, which enhances parasite transmission at the expense of mosquito fitness^74–76^. In contrast, in hard ticks of the genus *Ixodes*, each stage takes one blood meal from a single host, and there is no evidence that tick-borne pathogens can manipulate their tick vectors to take multiple blood meals. Following their acquisition during the larval blood meal, the interests of the tick-borne pathogen and the immature tick are largely the same, as both require the engorged larva to moult into a nymph, survive, and take a nymphal blood meal to complete their life cycles. One exception is when the infected *Ixodes* nymph chooses a vertebrate host that is not competent to harbour the tick-borne pathogen^29^. Conflict can also occur between the vector-borne pathogen and the arthropod vector if re-allocation of energetic resources in the arthropod vector from reproduction to somatic function (e.g., survival, searching for a host) enhances pathogen transmission^9,77,78^. Although female nymphs and male nymphs are different (the former take a much larger nymphal blood meal than the latter), it is unknown how much energy from the larval blood meal (if any) is allocated to sexual development in the unfed nymph. However, from the perspective of *B. burgdorferi* sl, which has no vertical transmission^79–81^, all energy from the larval blood meal should be devoted to finding a host and none should be wasted on sexual development in the nymph. Whether this conflict over tick sexual development between ticks and their tick-borne pathogens actually occurs remains unknown. In summary, there are more reports of adaptive manipulation for insect-borne pathogens compared to tick-borne pathogens^8^ and one explanation is that the life cycle of hard ticks is not very conducive to manipulation because each stage is strongly selected to complete a single blood meal on a single host.

### Effect of microbiota dysbiosis on life history traits of *I. ricinus*

Our study found that egg surface sterilization (with 10% bleach and 70% ethanol) at 28 days post-laying was highly effective at reducing the bacterial microbiota in the resultant *I. ricinus* larvae. This result agrees with similar studies on insects that used egg surface sterilization to manipulate the microbiota of the emerging insect larvae^54–58^. In insects, smearing bacteria over the surface of newly deposited eggs is a common extracellular route of transferring symbionts from mothers to their offspring^82^. Egg surface sterilization, by killing the bacteria on the surface of the egg, prevents the newly hatched larvae from acquiring these micro-organisms in either their gut or on their cuticle. Our study found that the abundance of the bacterial microbiota (as measured by the *16S rRNA* gene copy number) in the *I. ricinus* larvae at 6 weeks after hatching was 28 times lower in the surface-sterilized group compared to the control group. In a previous study on the same ticks, analysis of the *16S rRNA* gene diversity suggested that the egg surface sterilization treatment also changed the composition of the larval microbiota^51^. For example, the relative abundance of bacteria presumably associated with the egg surface (e.g., *Pseudomonas*) decreased, whereas the relative abundance of endosymbiotic bacteria (e.g., *Candidatus* Midichloria mitochondrii) increased^51^. However, our study cannot tell us whether the egg surface sterilization treatment changed the microbiota of the *I. ricinus* larvae in the gut, on the cuticle, or elsewhere.

Despite the strong reduction of the larval microbiota, the egg surface sterilization treatment did not have any effect on the life history traits of the immature *I. ricinus* ticks. This result contradicts a study that manipulated the microbiota of *I. scapularis* larvae by letting engorged females lay their eggs in sterile tubes^25^. In that study, dysbiosed *I. scapularis* larvae took larger blood meals than control larvae^25^. Our results are comparable to studies on *I. ricinus* and *I. pacificus* that found that injection of antibiotics into engorged female ticks had no effect on their reproductive fitness^38,39^. In other tick species that carry nutritional bacterial symbionts, such as *Coxiella*-like endosymbionts and *Francisella*-like endosymbionts that provide B vitamins, antibiotic treatments reduced tick fitness (e.g., moulting rate, body weight, fecundity, larval survival) and induced abnormal phenotypes ^15–20^. Normal tick phenotypes were recovered after combining the antibiotic treatment with an artificial blood meal containing the missing B vitamins^19,83,84^. Egg surface sterilization is not expected to have an impact on these *Coxiella*-like and *Francisella*-like endosymbionts because these obligate intracellular bacteria are exclusively transmitted through the egg cytoplasm^19,83,84^. In summary, a successful and dramatic reduction of the larval microbiota via egg surface sterilization had no meaningful impact on the life history traits of immature *I. ricinus* ticks.

### Density-dependent mortality of *I. ricinus*

We found evidence for density-dependent mortality of immature *I. ricinus*. Mice were infested with a standardized number of *I. ricinus* larvae, but differences in larval attachment and/or mouse grooming behaviour^85^ led to variation in the number of engorged larvae that were recovered from the mice. There was a strong negative correlation between the number of engorged larvae that were recovered from each mouse and the probability of immature tick survival. Density-dependent mortality occurs when the host develops anti-tick immunity so that the fitness of engorged larvae decreases over successive infestations^86–88^. However, the mice were only infested twice in the present study (once with nymphs and once with larvae), and the mechanism underlying this density-dependent mortality is therefore not clear. Nevertheless, density-dependent mortality of immature ticks on vertebrate hosts is an important mechanism regulating *Ixodes* tick populations^89–91^. We also found strong positive correlations between larva-to-nymph moulting time and the weights of the engorged larvae and unfed nymphs. This result suggests that larger larval blood meals take longer to digest, which increases the duration of the larva-to-nymph moult but results in a larger unfed nymph. The effects were relatively modest; for example, two engorged larvae with weights of 400 μg and 500 μg will moult in 40.5 days and 46.3 days, respectively. Finally, we found a strong correlation between the weights of the engorged larvae and the weights of the unfed nymphs. This result shows that larvae that take bigger blood meals moult into larger nymphs, which presumably have more resources for questing and survival in the field^28,34^. This result also shows that our measurements of engorged larval weight and unfed nymphal weight, which were done independently and separated by a time interval of 10 weeks, are highly reliable.

### Effects of tick family and mouse ID

We found highly significant effects of tick family and mouse ID on most of the life history traits of the immature *I. ricinus* ticks. Variance component analysis showed that tick family and mouse ID together account for 14% and 12% in the total variance of engorged larval weight and unfed nymphal weight, respectively. The factors underlying differences among tick families include genetics, nutritional condition of the mother tick, quality of the blood meal, microbiome of the mother tick, clutch size, and other factors. Similarly, even though the BALB/c mice were acquired from a commercial breeder, they still differ in body size, nutritional condition, skin microbiota, stress levels, and other factors. Regardless of the reasons for the phenotypic variation among larvae from different families and among larvae that fed on different BALB/c mice, studies that do not account for these sources of variation can produce biased results. For the statistical analysis of these data, it is essential to use mixed effects models that estimate and thereby control the variance in tick phenotype that is due to tick family and mouse ID.

## Conclusion

In summary, we found that infection with *B. afzelii* reduced the larva-to-nymph moulting time of engorged *I. ricinus* larvae, but the effect was small (2.4% reduction) and unlikely to be biologically significant. We found no evidence that infection with *B. afzelii* influenced the four other life history traits of immature *I. ricinus* ticks including engorged larval weight, unfed nymphal weight, larva-to-nymph moulting success, and immature tick survival. Although the egg surface sterilization treatment was highly effective at reducing the abundance of the bacterial microbiota in *I. ricinus* larvae, there was no evidence that this dysbiosis influenced the five life history traits of the immature *I. ricinus* ticks. Under the environmental conditions of this study, we conclude that *B. afzelii* and the egg-associated microbiota had no meaningful effect on tick fitness and hence on the R_0_ of Lyme borreliosis.

## Supplementary Information

Supplementary Information.

## Data Availability

The raw data for this study will be provided upon request to the corresponding author.
