# Publisher Correction: Infection with *Borrelia afzelii* and manipulation of the egg surface microbiota have no effect on the fitness of immature *Ixodes ricinus* ticks

**DOI:** 10.1038/s41598-021-93066-2

**Published:** 2021-07-13

**Authors:** Georgia Hurry, Elodie Maluenda, Anouk Sarr, Alessandro Belli, Phineas T. Hamilton, Olivier Duron, Olivier Plantard, Maarten J. Voordouw

**Affiliations:** 1 Department of Veterinary Microbiology, Western College of Veterinary Medicine, University of Saskatchewan, Saskatoon, Canada; 2 Laboratory of Ecology and Evolution of Parasites, Institute of Biology, University of Neuchâtel, Neuchâtel, Switzerland; 3 Laboratory of Ecology and Epidemiology of Parasites, Institute of Biology, University of Neuchâtel, Neuchâtel, Switzerland; 4 Deeley Research Centre, BC Cancer, Victoria, BC Canada; 5 Centre of Research in Ecology and Evolution of Diseases (CREES), Montpellier, France; 6 MIVEGEC (Maladies Infectieuses et Vecteurs: Ecologie, Génétique, Evolution et Contrôle), University of Montpellier (UM)-Centre National de la Recherche Scientifique (CNRS)-Institut Pour la Recherche et le Développement (IRD), Montpellier, France; 7 INRAE, Oniris, BIOEPAR, Nantes, France

Correction to: *Scientific Reports * 10.1038/s41598-021-90177-8, published online 21 May 2021

The original version of this Article contained errors.

Firstly, Affiliation 7 was incorrectly given as ‘INRAE, Oniris, BIOEPAR, 44300 Nantes, France’. The correct affiliation is listed below:

INRAE, Oniris, BIOEPAR, Nantes, France.

Secondly, the section ‘Approval for animal experiments’ was a duplication of the section ‘Approval for the use of experimental animals in the study’ in the Materials and methods and was subsequently removed.

Thirdly, in the Results section, under the subheadings ‘Prevalence of *B. afzelii* infection in unfed *I. ricinus* nymphs', ‘Relationship between engorged larval weight and unfed nymphal weight’ and ‘Power analysis to determine the minimal detectable effect size.’

“The 3 nymphs that fed had as larvae on the uninfected control mice, but that tested positive for *B. afzelii* on the qPCR are false positives (these 3 nymphs had high Cq values of 39.62, 39.74, and 40.22; see Section 5 of the ESM).”

now reads:

“The 3 nymphs that had fed as larvae on the uninfected control mice, but that tested positive for *B. afzelii* on the qPCR are false positives (these 3 nymphs had high Cq values of 39.62, 39.74, and 40.22; see Section 5 of the ESM).”

“To test this hypothesis, we analyzed the probability that the *I. ricinus*larvae acquired *B. afzelii* as a function of the size of the larval blood meal for the subset of *I. ricinus* larvae that fed on the 20 infected mice.”

now reads:

“To test this hypothesis, we analyzed the probability that the *I. ricinus* larvae acquired *B. afzelii* as a function of the size of the larval blood meal for the subset of larvae that fed on the 20 infected mice.”

“For three life history traits (moulting time, engorged larval weight, and unfed nymphal weight) our sampling effort had a power > 80% to detect a treatment effect (of *B. afzelii* infection or egg surface sterilization) that either increased or decreased tick phenotype by ~ 5% (relative to the control).”

now reads:

“For three life history traits (moulting time, engorged larval weight, and unfed nymphal weight) our sampling effort had a power > 80% to detect a treatment effect (of *B. afzelii* infection or egg surface sterilization) that either increased or decreased the tick phenotype by ~ 5% (relative to the control).”

Fourthly, in the legend of Figure 5,

“*I. ricinus* larvae that feed on *B. afzelii*-infected mice have a faster larva-to-nymph moulting time compared to larvae that feed on uninfected control mice.”

now reads:

“*Ixodes* *rinicus* larvae that feed on *B. afzelii*-infected mice have a faster larva-to-nymph moulting time compared to larvae that feed on uninfected control mice.”

Fifthly, in the Discussion, under the subheadings ‘Importance of controlled experimental infections’ and ‘Scope of selection for manipulation of arthropod vectors by vector‑borne pathogens.’

“Our study shows the importance of using controlled experiments to test whether infection with tick-borne pathogens sl influences the life history traits of their tick vectors.”

now reads:

“Our study shows the importance of using controlled experiments to test whether infection with tick-borne pathogens influences the life history traits of their tick vectors.”

“Although female nymphs and male nymphs are different (the former takes a much larger nymphal blood meal than the latter), it is unknown how much energy from the larval blood meal (if any) is allocated to sexual development in the unfed nymph.”

now reads:

“Although female nymphs and male nymphs are different (the former take a much larger nymphal blood meal than the latter), it is unknown how much energy from the larval blood meal (if any) is allocated to sexual development in the unfed nymph.”

Finally, References 1, 8, 11, 39, 41, 70, 71, 77, 83 and 86 were incorrectly given as:

1. Randolph, S. E. Ticks are not insects: Consequences of contrasting vector biology for transmission potential. *Parasitol Today* **14**, 186–192 (1998).

8. Lefevre, T. & Thomas, F. Behind the scene, something else is pulling the strings: Emphasizing parasitic manipulation in vector-borne diseases. *Infect. Genet. Evol.* **8**, 504–519. https://doi.org/10.1016/j.meegid.2007.05.008 (2008).

11. Benelli, G. Pathogens manipulating tick behavior-through a glass. *Darkly. Pathogens* https://doi.org/10.3390/pathogens9080664 (2020).

39. Ninio, C. et al*.* Antibiotic treatment of the hard tick Ixodes ricinus: Influence on *Midichloria mitochondrii* load following blood meal. *Ticks Tick Borne Dis.* **6**, 653–657. https://doi.org/10.1016/j.ttbdis.2015.05.011 (2015).

41. Telford, S. R., Mather, T. N., Moore, S. I., Wilson, M. L. & Spielman, A. Incompetence of deer as reservoirs of the Lyme-disease spirochete. *Am. J. Trop. Med. Hyg.* **39**, 105–109 (1988).

70. Gray, J. S. The development and seasonal activity of the tick Ixodes ricinus: a vector of Lyme borreliosis. *Rev. Med. Vet. Entomol.* **79**, 323–333 (1991).

71. Jouda, F., Perret, J. L. & Gern, L. *Ixodes ricinus* density, and distribution and prevalence of *Borrelia burgdorferi sensu lato* infection along an altitudinal gradient. *J. Med. Entomol.* **41**, 162–169. https://doi.org/10.1603/0022-2585-41.2.162 (2004).

77. Hurd, H. Host fecundity reduction: a strategy for damage limitation? Trends Parasitol. 17, 363–368 (2001).

83. Buysse, M., Plantard, O., McCoy, K. D., Duron, O. & Menard, C. Tissue localization of *Coxiella*-like endosymbionts in three European tick species through fluorescence in situ hybridization. *Ticks Tick Borne Dis.* **10**, 798–804. https://doi.org/10.1016/j.ttbdis.2019.03.014 (2019).

86. Randolph, S. E. Population regulation in ticks—Role of acquired-resistance in natural and unnatural hosts. *Parasitology* **79**, 141–156 (1979).

The correct references are listed below:

1. Randolph, S. E. Ticks are not insects: Consequences of contrasting vector biology for transmission potential. *Parasitol Today* **14**, 186–192 (1998).

8. Lefèvre, T. & Thomas, F. Behind the scene, something else is pulling the strings: Emphasizing parasitic manipulation in vector-borne diseases. *Infect. Genet. Evol.* **8**, 504–519. https://doi.org/10.1016/j.meegid.2007.05.008 (2008).

11. Benelli, G. Pathogens manipulating tick behavior-through a glass, darkly. *Pathogens* https://doi.org/10.3390/pathogens9080664 (2020).

39. Ninio, C. et al*.* Antibiotic treatment of the hard tick *Ixodes ricinus*: Influence on *Midichloria mitochondrii* load following blood meal. *Ticks Tick Borne Dis.* **6**, 653–657. https://doi.org/10.1016/j.ttbdis.2015.05.011 (2015).

41. Telford, S. R., Mather, T. N., Moore, S. I., Wilson, M. L. & Spielman, A. Incompetence of deer as reservoirs of the Lyme disease spirochete. *Am. J. Trop. Med. Hyg.* **39**, 105–109 (1988).

70. Gray, J. S. The development and seasonal activity of the tick *Ixodes ricinus*: a vector of Lyme borreliosis. *Rev. Med. Vet. Entomol.*** 79**, 323–333 (1991).

71. Jouda, F., Perret, J. L. & Gern, L. *Ixodes ricinus* density, and distribution and prevalence of *Borrelia burgdorferi* sensu lato infection along an altitudinal gradient. *J. Med. Entomol.* **41**, 162–169. https://doi.org/10.1603/0022-2585-41.2.162 (2004).

77. Hurd, H. Host fecundity reduction: a strategy for damage limitation? *Trends Parasitol*.** 17**, 363–368 (2001).

83. Buysse, M., Plantard, O., McCoy, K. D., Duron, O. & Menard, C. Tissue localization of *Coxiella*-like endosymbionts in three European tick species through fluorescence* in situ* hybridization. *Ticks Tick Borne Dis.* **10**, 798–804. https://doi.org/10.1016/j.ttbdis.2019.03.014 (2019).

86. Randolph, S. E. Population regulation in ticks: the role of acquired resistance in natural and unnatural hosts. *Parasitology* **79**, 141–156 (1979).

The original Article has been corrected.

