# Supplementary material for: Infection with Borrelia afzelii and manipulation of the egg surface microbiota have no effect on the fitness of immature Ixodes ricinus ticks
Source: Sci Rep. 2021 May 21;11:10686. doi: 10.1038/s41598-021-90177-8 (PMC8140075; doi:10.1038/s41598-021-90177-8)
Supplement: Supplementary file 1 — Supplementary Information. [file 41598_2021_90177_MOESM1_ESM.docx]

Supplementary Material

Title: Infection with *Borrelia afzelii* and manipulation of the egg surface microbiota have no effect on the fitness of immature *Ixodes ricinus* ticks

Authors: Georgia Hurry, Elodie Maluenda, Anouk Sarr, Alessandro Belli, Phineas T. Hamilton, Olivier Duron, Olivier Plantard, Maarten J. Voordouw

Table of Contents

[Section 1 – Pilot study on the surface sterilization treatment of I. ricinus eggs 2](#_Toc66273118)

[Section 2 – Surface sterilization treatment of I. ricinus eggs to create dysbiosed larvae 3](#_Toc66273119)

[Section 3 - Creation of experimentally infected I. ricinus nymphs 4](#_Toc66273120)

[Section 4 – Molecular methods for I. ricinus nymphs 4](#_Toc66273121)

[Section 5 - B. afzelii infection prevalence of Ixodes ricinus nymphs 5](#_Toc66273122)

[Section 6 - Host-to-tick transmission of B. afzelii 6](#_Toc66273123)

[Section 7 - Effect of B. afzelii infection status of the mice on the conversion efficiency of the larval blood meal into the weight of unfed nymphs 7](#_Toc66273124)

[Section 8 – Size of larval blood meal and the probability of acquiring B. afzelii 8](#_Toc66273125)

[Section 9 - Effect of B. afzelii infection status of the mice and egg washing treatment on the weight of the immature I. ricinus ticks 8](#_Toc66273126)

[Section 10 - Effect of B. afzelii infection status of the ticks and the egg bleaching treatment on the weight of the immature I. ricinus ticks 11](#_Toc66273127)

[Section 11 – Test of assumptions of the LMM and GLMMs 14](#_Toc66273128)

[Section 12 – Effects of tick family and mouse ID on the life history traits of immature I. ricinus ticks 15](#_Toc66273129)

[Section 13 – Power analysis to determine the minimal detectable effect size 17](#_Toc66273130)

# **Section 1 – Pilot study on the surface sterilization treatment of *I. ricinus* eggs**

The purpose of the pilot study was to develop a treatment that would allow us to reduce the microbiome in *Ixodes ricinus* larval ticks by surface sterilizing the eggs. Numerous studies on insects have manipulated the microbiome of larvae by sterilizing the surface of the eggs prior to hatching (Prado et al. 2006, Prado and Almeida 2009, Salem et al. 2013, Bistolas et al. 2014, Taylor et al. 2014). Most of these studies submerse the insect eggs in solutions of bleach (10%) and/or ethanol (95% to 100%) for short periods of time (5 to 7 minutes). For the pilot experiment, we obtained 10 engorged *I. ricinus* females that had been collected from roe deer in the Chizé forest. For egg laying, the engorged females were placed in sterilized glass vials with screw caps that contained a piece of moistened Whatman filter paper to maintain a high relative humidity. These glass vials were placed in a phytotron with the following conditions: a 16-hour light phase with a temperature of 25 °C followed by an 8-hour dark phase with a temperature of 18 °C. There was a 1-hour phase at dawn and dusk where the temperature was 21.5°C.

The 10 females laid eggs between 15 February and 22 February 2016. The clutches of the 10 females were divided into 5 equal batches. To facilitate submersion of the eggs in the different sterilization solutions, each of the 50 batches of eggs was placed in a cell strainer with a mesh of 100 μm. These batches of eggs were exposed to 10 different egg surface sterilization treatments (treatments A to L in Table S1). In all the egg surface sterilization treatments, the eggs were submersed in a bleach solution and in 70% ethanol followed by a rinse with distilled water (dH_2_O). For the bleach step, the concentration of the bleach solution was either 5% or 10% and the duration of the immersion was either 0.5 min, 1.5 min, or 5.0 min. For the ethanol step, the concentration was always 70% and the duration was always 3 min. In addition to the 10 egg surface sterilization treatments, there were also two control treatments where the eggs were left alone (treatment M) or where the eggs were submersed in dH_2_O for 5 min, submersed in dH_2_O for 3 min, and then rinsed with dH_2_O (treatment N). The eggs were rinsed from the cell strainer onto a piece of Whatman filter paper that was left to dry inside a biosafety cabinet at room temperature. The dried eggs and filter paper were placed inside a glass petri dish that was sealed with parafilm to prevent the larvae from escaping. This glass petri dish was placed inside the phytotron under the same conditions as mentioned previously.

We monitored the hatching success of the eggs over a period of 50 days. The hatching success of the larvae was very high (~100%) and there were no obvious differences in the hatching success between the 10 egg surface sterilization treatments and the two control treatments. For our main study, we therefore chose the most extreme egg surface sterilization treatment, which consisted of submersion in 10% bleach for 5 min, submersion in 70% ethanol for 3 min, and rinsing with dH_2_O (treatment F). To match the submersion times of the egg surface sterilization treatment, the control treatment consisted of submersion in dH_2_O for 5 min, submersion in dH_2_O for 3 min, and rinsing with dH_2_O (treatment N).

Table S1. The egg surface sterilization treatments in the pilot study

| Treatment | Step 1 | Step 2 | Step 3 | Female ID |
| --- | --- | --- | --- | --- |
| A | 5% bleach for 0.5 min | 70% ethanol for 3 min | Rinse with dH2O | 7, 8 |
| B | 5% bleach for 1.5 min | 70% ethanol for 3 min | Rinse with dH2O | 7, 8 |
| C | 5% bleach for 5.0 min | 70% ethanol for 3 min | Rinse with dH2O | 7, 8 |
| D | 10% bleach for 0.5 min | 70% ethanol for 3 min | Rinse with dH2O | 1, 2, 3 |
| E | 10% bleach for 1.5 min | 70% ethanol for 3 min | Rinse with dH2O | 1, 2, 3 |
| F | 10% bleach for 5.0 min | 70% ethanol for 3 min | Rinse with dH2O | 1, 2, 3 |
| G | 70% ethanol for 3 min | 5% bleach for 0.5 min | Rinse with dH2O | 9, 10 |
| H | 70% ethanol for 3 min | 5% bleach for 1.5 min | Rinse with dH2O | 9, 10 |
| I | 70% ethanol for 3 min | 5% bleach for 5.0 min | Rinse with dH2O | 9, 10 |
| J | 70% ethanol for 3 min | 10% bleach for 0.5 min | Rinse with dH2O | 4, 5, 6 |
| K | 70% ethanol for 3 min | 10% bleach for 1.5 min | Rinse with dH2O | 4, 5, 6 |
| L | 70% ethanol for 3 min | 10% bleach for 5.0 min | Rinse with dH2O | 4, 5, 6 |
| M | NA | NA | NA | all |
| N | dH2O for 5 min | dH2O for 3 min | Rinse with dH2O | all |

# **Section 2 – Surface sterilization treatment of *I. ricinus* eggs to create dysbiosed larvae**

On 15 March 2016, 11 engorged *I. ricinus* females were collected from roe deer in the Chizé forest. On 24 March 2016, these engorged females arrived at the University of Neuchatel. For egg laying, the engorged females were placed in sterilized glass vials with screw caps that contained a piece of moistened Whatman filter paper to maintain a high relative humidity. These glass vials were placed in a phytotron with the following conditions: a 16-hour light phase with a temperature of 25 °C followed by an 8-hour dark phase with a temperature of 18 °C. There was a 1-hour phase at dawn and dusk where the temperature was 21.5°C. One of the 11 females died, but the remaining 10 females started to lay their eggs between 15 April and 20 April 2016. At 28 days after oviposition, the eggs were exposed to the egg surface sterilization treatment (treatment F in Table 1) or the control treatment (treatment N in Table S1). Each of the 10 egg masses was divided into two equal batches. To facilitate submersion of the eggs in the different sterilization solutions, each of the 20 batches of eggs was placed in a cell strainer with a mesh of 100 μm. In the egg surface sterilization treatment, the eggs were submersed in a 10% bleach solution for 5 minutes, a 70% ethanol solution for 3 minutes, before being rinsed gently with distilled water for 3 minutes (treatment F in Table S1). In the control treatment, the 10% bleach and 70% ethanol solutions were replaced with distilled water (treatment N in Table S1). The eggs were rinsed from the cell strainer onto a piece of Whatman filter paper that was left to dry inside a biosafety cabinet at room temperature. The dried eggs and filter paper were placed inside a glass petri dish that was sealed with parafilm to prevent the larvae from escaping. This glass petri dish was placed inside the phytotron under the same conditions as mentioned previously. We monitored the hatching success of the eggs and at 50 days after the egg washing treatment ~100% of the larvae had hatched. As before in the pilot study, there were no obvious differences in hatching success between the eggs exposed to the egg surface sterilization treatment versus the control treatment.

# **Section 3 - Creation of experimentally infected *I. ricinus* nymphs**

BALB/c mice were needle-inoculated with *B. afzelii* strain NE4049. Five weeks following inoculation, larval ticks from our pathogen-free laboratory colony of *Ixodes ricinus* were allowed to feed to repletion on the infected mice. The engorged larvae were placed in individual Eppendorf tubes and allowed to moult into nymphs. The proportion of *I. ricinus* nymphs infected with *B. afzelii* isolate NE4049 ranged between 80.0% and 100.0%. In addition, larval ticks were fed on uninfected BALB/c mice to create uninfected control nymphs.

# **Section 4 – Molecular methods for *I. ricinus* nymphs**

***Borrelia flagellin* gene qPCR:** The total spirochete load of *Borrelia afzelii* in the *Ixodes ricinus* nymphs was estimated using a qPCR that targeted a 132-bp fragment of the *flagellin* gene (Schwaiger et al. 2001). The qPCRs were performed using the LightCycler^®^ 480 Multiwell Plate 96 white (Roche). The wells were filled with a mixture of 5.8 µl of water, 10.0 µl of Master Mix (FastStart Essential DNA probes Master, Roche), 0.4 µl of 20.0 µM forward primer FlaF1A, 0.4 µl of 20.0 µM reverse primer FlaR1, 0.4 µl of 10.0 µM Flaprobe1, and 3.0 µl of DNA template. The thermocycling conditions consisted of 10 min at 95°C for denaturation, followed by 50 cycles of 30 sec at 60°C and 10 sec at 95°C.

**Bacterial *16S rRNA* gene qPCR**: We quantified bacterial load in each tick using a SYBR Green real-time qPCR assay that amplified the V3 hypervariable region of the 16S rRNA gene. The primers used were 338f (5’-ACTCCTACGGGAGGCAGCAG-3’) and 520r (5’-ATTACCGCGGCTGCTGG-3’) (Muyzer et al. 1993, Bakke et al. 2011). The qPCR was based on a previously developed protocol (Bueche et al. 2013). The qPCRs were carried out in a final reaction volume of 10 µl with 5 µl Rotor-Gene SYBR green PCR master mix (Qiagen GmbH, Hilden, Germany), 0.30 µM of forward primer 338f, 0.30 µM of reverse primer 520r, and and 3.0 µl of DNA template. The thermocycling conditions consisted of an initial denaturation/activation step at 95°C for 15 min, followed by 40 cycles composed of denaturation at 95°C for 10 s, annealing at 55°C for 15 s, and elongation at 72°C for 20 s. Each 96-well qPCR plate contained 5 standards, and 3 negative controls that were all run in triplicate. The five standards contained 10^7^, 10^6^, 10^5^, 10^4^ and 10^3^ copies of the *16s rRNA* gene.

Standards were produced using a plasmid containing the *16s rRNA* gene sequence (Bueche et al. 2013). Plasmid-transformed *E. coli* cells were grown up overnight and plasmid DNA was extracted with the Wizard Plus SV Miniprep DNA purification system (Promega, Switzerland) following the manufacturer’s instructions. The DNA concentration of the plasmid mini prep was estimated using a Nanodrop 2000 (Thermo Scientific) and the number of 16s rRNA gene copies was calculated using the known molecular weight of the plasmid (3326251.8 g/mol).

***Ixodes ricinus calreticulin* gene qPCR:** Variation in DNA extraction efficiency and DNA concentration in the DNA extractions will influence the estimates of the *flagellin* gene copy number and the *16S rRNA* gene copy number in the samples. To control for this variation, we standardized our estimates of bacterial abundance with respect to an estimate of the *I. ricinus* nuclear *calreticulin* gene copy number in the samples. We used a SYBR Green real-time qPCR assay that targeted a 109-bp fragment of the *calreticulin* (*cal*)gene as previously described (Sassera et al. 2008). The primers used were calF (5’- ATCTCCAATTTCGGTCCGGT -3’) and calR (5’- TGAAAGTTCCCTGCTCGCTT -3’) (Sassera et al. 2008). The qPCRs were carried out in a final reaction volume of 20 µl with 12.5 µl Rotor-Gene SYBR green PCR master mix (Qiagen GmbH, Hilden, Germany), 0.40 µM of forward primer calF, 0.40 µM of reverse primer calR, and and 3.0 µl of DNA template. PCR cycling conditions for the *calreticulin* gene were as follows: 95°C for 2 min, 40 cycles at 95°C for 15 s and at 60°C for 30 s, and melt curve analysis from 55°C to 95°C with increasing increments of 0.5°C per cycle.

Each 96-well qPCR plate contained 5 standards, and 3 negative controls that were all run in triplicate. The five standards contained 10^7^, 10^6^, 10^5^, 10^4^ and 10^3^ copies of the *calreticulin* gene. Standards were produced using a pGEMT-easy plasmid containing the *calreticulin* gene sequence. Plasmid-transformed *E. coli* cells were grown up overnight and plasmid DNA was extracted with the Wizard Plus SV Miniprep DNA purification system (Promega, Switzerland) following the manufacturer’s instructions. The DNA concentration of the plasmid mini-prep was estimated using a Nanodrop 2000 (Thermo Scientific) and the number of *calreticulin* gene copies was calculated using the known molecular weight of the plasmid.

**ELISA to determine whether mice developed IgG antibodies against *B. afzelii*:** We used the SERION ELISA classic *B. burgdorferi* sl IgG/IgM immunoassay to detect the presence of IgG antibodies against *B. afzelii*, as we have described previously (Belli et al. 2017). Briefly, the ELISA plate was incubated with the mouse serum samples diluted 1:100 in blocking solution (2% bovine serum albumin in phosphate-buffered saline (PBS)) for 45 min at room temperature. The ELISA plate was incubated with a goat anti-mouse IgG horseradish peroxidase conjugate diluted 1:5000 in blocking solution for 45 min at room temperature. After each incubation step, the plate was washed three times with a solution of 0.1% TWEEN in PBS for 5 min. After adding 100 μl of tetramethylbenzidine (TMB solution) to each well, the absorbance at a wavelength of 652 nm was measured every 2 min for 1 hour to quantify the IgG antibody response against *B. afzelii*. We used the area under the curve function, *auc()*, in R to calculate the total absorbance over the 60 min of the reaction.

# **Section 5 - *B. afzelii* infection prevalence of *Ixodes ricinus* nymphs**

We fed *I. ricinus* larvae on the uninfected control mice and on the *B. afzelii*-infected mice. The engorged larvae were allowed to moult into nymphs. We used a *flagellin* qPCR with 40 cycles to determine whether the nymphs had acquired *B. afzelii* during the larval blood meal. The Cq value is the cycle during the qPCR at which the sample crosses the minimum fluorescence threshold. Low and high Cq values correspond to samples with high and low amounts of the target gene. To determine whether there is a threshold Cq value that allows us to differentiate between infected and uninfected nymphs, we created a histogram of the Cq values for the subset of infected nymphs. This histogram of the Cq values of infected nymphs shows a normal-like distribution with no clear threshold separating infected and uninfected nymphs (**Figure S1**).

Of the nymphs that fed on the uninfected control mice, 2.8% (3/106) tested positive for *B. afzelii* infection. As we know that the mice were uninfected, we know that these 3 nymphs produced a false positive result. These 3 nymphs had the following Cq values: 39.62, 39.74, 40.22. These Cq values were in the top 9 largest Cq values, providing further support that these results are false positives. These findings suggest that a Cq threshold of ~39 differentiates (imperfectly) between infected and uninfected nymphs.

**Figure S1.** Frequency of the Cq values obtained from qPCR of the subset of *B. afzelii*-infected nymphal ticks.

# **Section 6 - Host-to-tick transmission of B. afzelii**

The percentage of nymphs that are infected with *B. afzelii* is an estimate of host-to-tick transmission (i.e., percentage of engorged larvae that acquired *B. afzelii* during the larval blood meal and that moulted into infected nymphs). For the subset of infected mice (n = 20), the host-to-tick transmission ranged from 44.4 – 90.0%, with a mean infection prevalence of 70.9% (**Figure S2**). For the uninfected control mice (n = 19), most of the mice (16/19) had host-to-tick transmission of 0.0%, whereas the 3 false positive nymphs mentioned earlier caused the remaining mice (3/19) to have host-to-tick transmission > 0.0% (**Figure S2**).

**Figure S2.** Host-to-tick transmission of *B. afzelii* for uninfected control mice versus infected mice. Host-to-tick transmission was measured as the percentage of nymphs that acquired *B. afzelii* (as shown by the *flagellin* qPCR) after feeding as larvae on the uninfected control mice versus the experimentally infected mice. The three nymphs that tested positive for *B. afzelii* (on the *flagellin* qPCR) after feeding on 3 different uninfected control mice are false positives. The boxplots show the median (black line), 25th and 75th percentiles (edges of the box), minimum and maximum values (whiskers), and outliers (open circles).

# **Section 7 - Effect of *B. afzelii* infection status of the mice on the conversion efficiency of the larval blood meal into the weight of unfed nymphs**

We created a new explanatory factor called ‘group’ with 4 levels for each of the 4 combinations of egg treatment and mouse infections status: (1) water and uninfected, (2) bleach and uninfected, (3) water and infected, and (4) bleach and infected. We modelled the log10-transformed unfed nymphal weight as a function of this ‘group’ factor and the log10-transformed larval weight as a covariate. The parameter estimates were used to generate regression equations. In these regression equations, X = log10-transformed engorged larval weight and Y = log10-transformed unfed nymphal weight.

1. Water and Uninfected: Y = -0.06705 + 0.87615*X
2. Bleach and Uninfected: Y = -0.27455 + 0.95408*X
3. Water and Infected: Y = -0.27662 + 0.95691*X
4. Bleach and Infected: Y = -0.01952 + 0.85937*X

These equations allow for the prediction of unfed nymphal weight from engorged larval weight. The slope is a measure of the efficiency by which the larval blood meal is converted into the weight of the flat nymph. However, we found no evidence that this slope differed between the four groups.

# **Section 8 – Size of larval blood meal and the probability of acquiring *B. afzelii***

**Statistical Methods:** A previous study had shown that *I. scapularis* larvae that took larger blood meals had a higher probability to acquire *B. burgdorferi* sensu stricto (ss) (Couret et al. 2017). GLMMs with binomial errors were used to test whether the infection status of the flat nymphs (response variable) depended on the size of the larval blood meal (explanatory variable), as measured by the engorged larval weight (transformed to z-scores). This analysis was restricted to the subset of 177 ticks that fed as larvae on the *B. afzelii*-infected mice, and for which we obtained both the engorged larval weight and the infection status in the resultant unfed nymph.

**Results:** To test whether the amount of blood ingested during the larval blood meal (as measured by the engorged larval weight) influenced the infection status of the resultant unfed nymph (0 = uninfected, 1 = infected), we used a GLMM with binomial errors to model the latter as a function of the former. This analysis was done on the subset of 177 ticks that fed on the *B. afzelii*-infected mice and for which we obtained both the engorged larval weight and the infection status of the resultant unfed nymph. There was no effect of the engorged larval weight on the infection status of the resultant unfed nymph (slope = 0.095, std. error = 0.173, z = 0.550, p = 0.582). In summary, the size of the larval blood meal did not influence the probability that the tick acquired *B. afzelii* infection (as tested in the resultant unfed nymph).

# **Section 9 - Effect of *B. afzelii* infection status of the mice and egg washing treatment on the weight of the immature *I. ricinus* ticks**

We found no significant effects of *B. afzelii* infection in mice, egg washing treatment, or their interaction on the engorged larval weights (**Table S2**; **Figure S3**). Similarly, we found no significant effects of *B. afzelii* infection in mice, egg washing treatment, or their interaction on the unfed nymphal weights (**Table S2**; **Figure S4**). The statistical analyses of the engorged larval weights and the unfed nymphal weights are shown in the text of the main manuscript.

**Table S2.** Means and 95% confidence intervals of the weights of the engorged larvae and the weights of the unfed nymphs (ug) for each of the four combinations of egg washing treatment and mouse infection status. LL and UL refer to the lower limit (2.5% percentile) and the upper limit (97.5% percentile) of the 95% confidence interval of the mean. The parameter estimates were taken from the LMM of the log10-transformed weights.

| **Stage** | **Egg Treatment** | **Mouse Infection Status** | **N** | **Mean weight (ug)** | **LL** | **UL** |
| --- | --- | --- | --- | --- | --- | --- |
| Larva | Water | Uninfected | 180 | 450.0 | 433.2 | 467.7 |
| Larva | Bleach | Uninfected | 185 | 440.9 | 424.6 | 457.8 |
| Larva | Water | Infected | 179 | 443.1 | 426.4 | 460.4 |
| Larva | Bleach | Infected | 198 | 442.6 | 426.5 | 459.3 |
|  |  |  |  |  |  |  |
| Nymph | Water | Uninfected | 260 | 182.4 | 176.0 | 189.1 |
| Nymph | Bleach | Uninfected | 269 | 177.9 | 171.7 | 184.2 |
| Nymph | Water | Infected | 260 | 177.9 | 171.6 | 184.4 |
| Nymph | Bleach | Infected | 278 | 179.5 | 173.4 | 185.8 |

**
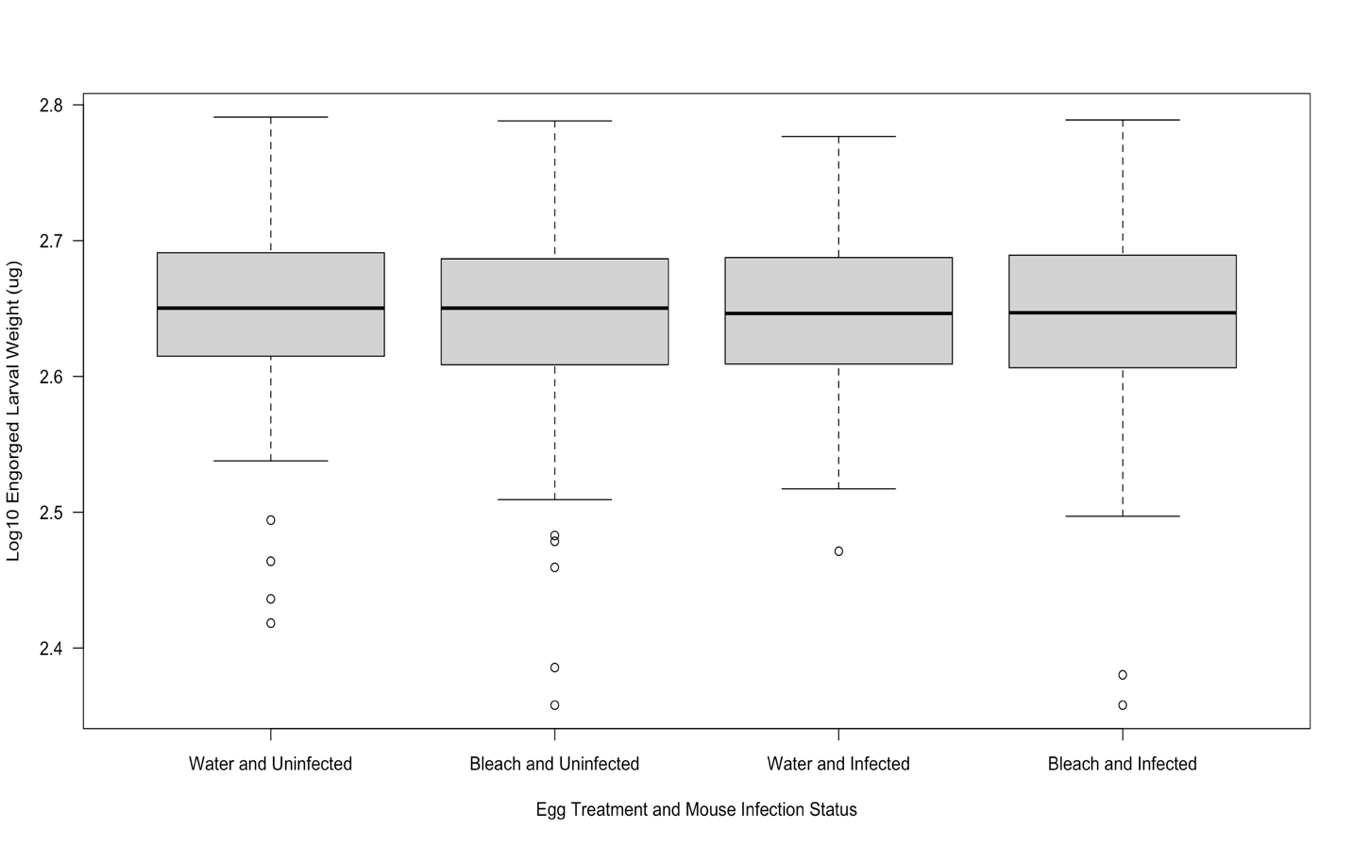
**

**Figure S3.** The *B. afzelii* infection status of the mouse has no effect on the weight of the engorged larvae that fed on these mice. Log10-transformed weights of engorged larval ticks (μg) are shown for each of the four combinations of egg washing treatment and mouse infection status. The boxplots show the median (black line), 25th and 75th percentiles (edges of the box), minimum and maximum values (whiskers), and outliers (open circles).

**
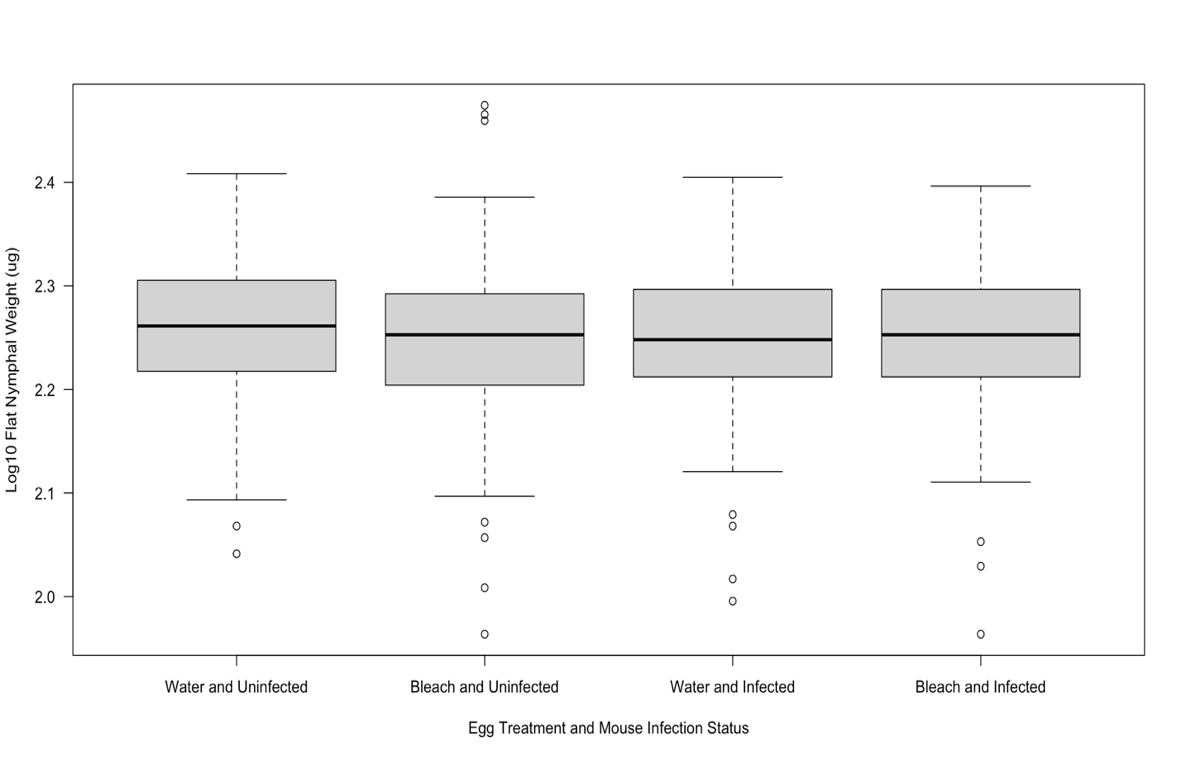
**

**Figure S4.** The *B. afzelii* infection status of the mouse has no effect on the weight of the unfed nymphs that fed on these mice during their larval stage. Log10-transformed weights of the unfed nymphal ticks (μg) are shown for each of the four combinations of egg washing treatment and mouse infection status. The boxplots show the median (black line), 25th and 75th percentiles (edges of the box), minimum and maximum values (whiskers), and outliers (open circles).

# **Section 10 - Effect of *B. afzelii* infection status of the ticks and the egg bleaching treatment on the weight of the immature *I. ricinus* ticks**

In the main manuscript, the *B. afzelii* infection status was treated as a state of the mouse. In other words, we compared the phenotype between ticks that had taken their larval blood meal from either a *B. afzelii*-infected mouse or an uninfected control mouse. However, not all *I. ricinus* larvae that feed on an infected mouse acquire the infection. For a subset of ticks, we determined the *B. afzelii* infection status of the ticks themselves. This tick infection status contained 3 types: (1) control ticks that had fed on uninfected mice (Control), (2) ticks that had fed on infected mice and that had acquired the infection (Infected), (3) ticks that had fed on infected mice and that had not acquired the infection (Uninfected). When these 3 types of ticks were combined with the egg washing treatment (Bleach versus Water), there were 6 unique combinations. We created boxplot to compare the engorged larval weight (**Figure S5**) and the unfed nymphal weight (**Figure S6**) among these 6 groups of ticks.

We found no significant effects of *B. afzelii* infection status of the ticks, egg washing treatment, or their interaction on the engorged larval weights (**Table S3**; **Figure S5**). Similarly, we found no significant effects of *B. afzelii* infection status of the ticks, egg washing treatment, or their interaction on the unfed nymphal weights (**Table S3**; **Figure S6**).

**Table S3.** Statistical analyses of the effect of *B. afzelii* infection status of the ticks on the engorged larval weight and unfed nymphal weight. LMMs were used to test the effects of mouse infection status (I), egg washing (E), and their interaction (I:E) on the two response variables. Shown are the results from the type II log-likelihood ratio test to determine the statistical significance of the explanatory variables. The row headers refer to the name of the response variable, the name of the explanatory variable, the degrees of freedom (df), the Chi-square statistic (χ^2^), and the p-value (p).

| **Response Variable** | **Explanatory variable** | **Df** | **χ^2^** | **p** |
| --- | --- | --- | --- | --- |
| Engorged larval weight | I:E interaction | 2 | 0.059 | 0.971 |
| Engorged larval weight | Tick infection status | 2 | 0.618 | 0.734 |
| Engorged larval weight | Egg washing | 1 | 0.146 | 0.703 |
|  |  |  |  |  |
| Unfed nymphal weight | I:E interaction | 2 | 0.177 | 0.916 |
| Unfed nymphal weight | Tick infection status | 2 | 0.989 | 0.610 |
| Unfed nymphal weight | Egg washing | 1 | 0.029 | 0.864 |

**
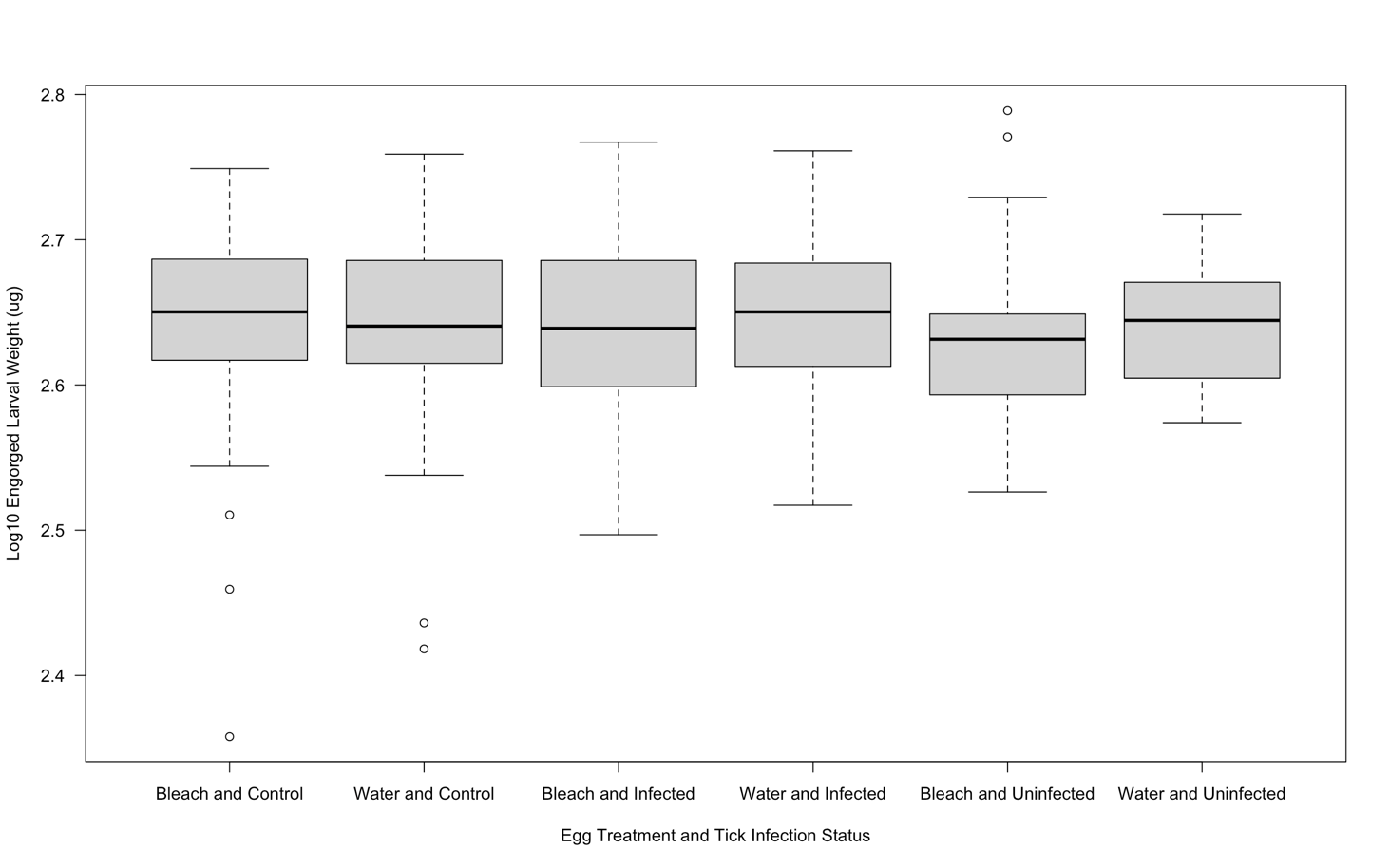
**

**Figure S5.** The *B. afzelii* infection status of the tick has no effect on the weight of the larvae that engorged on the control and infected mice. Log10-transformed weights of engorged larval ticks (ug) are shown for each of the six combinations of egg washing treatment and tick infection status. Uninfected ticks are those that fed on infected mice but did not acquire an infection. The boxplots show the median (black line), 25th and 75th percentiles (edges of the box), minimum and maximum values (whiskers), and outliers (open circles).

**
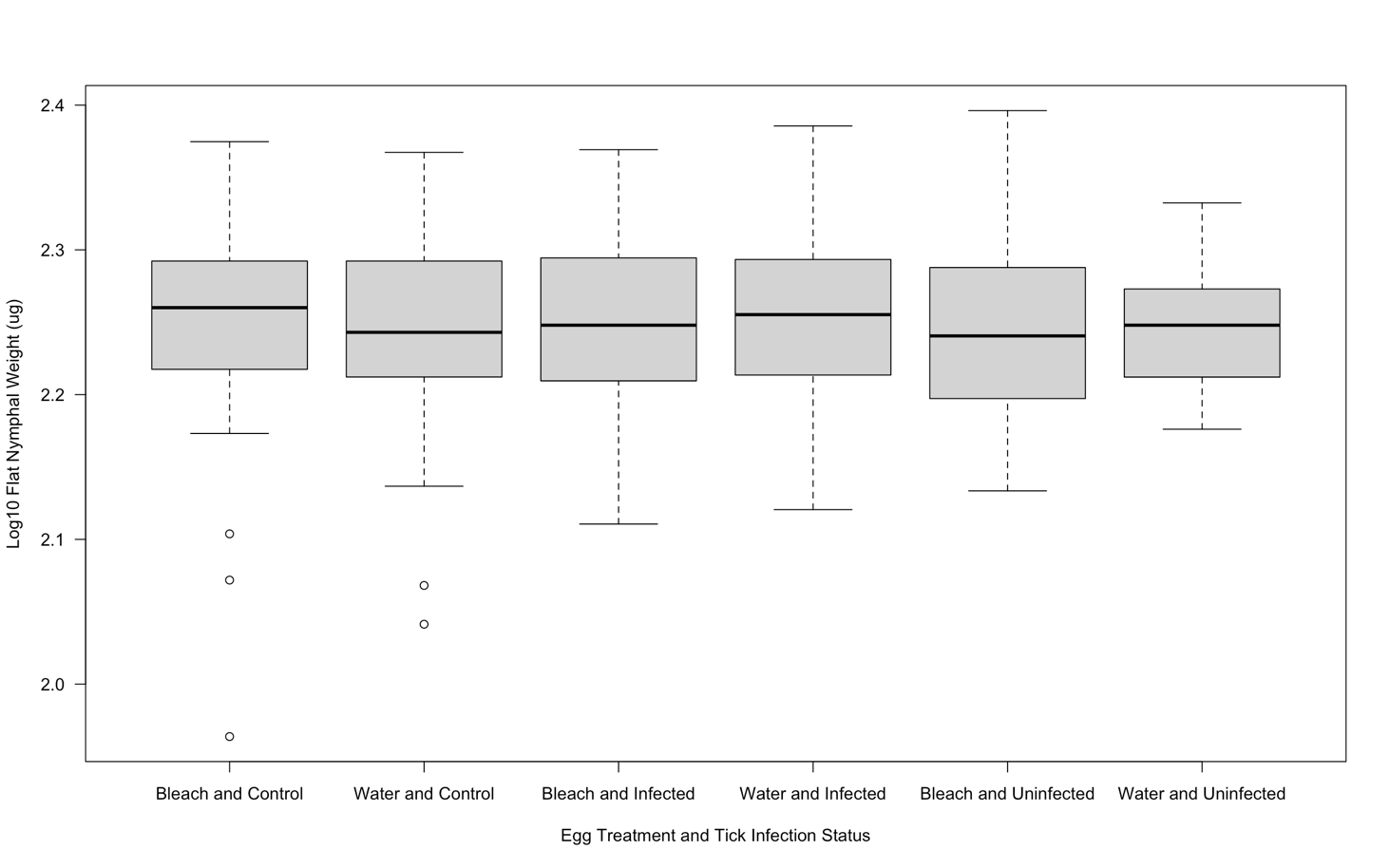
**

**Figure S6.** The *B. afzelii* infection status of the mouse has no effect on the weight of the flat nymphs that fed on the control and infected mice during their larval stage. Log10-transformed weights of engorged larval ticks (ug) are shown for each of the six combinations of egg washing treatment and tick infection status. Uninfected ticks are those that fed on infected mice but did not acquire an infection. The boxplots show the median (black line), 25th and 75th percentiles (edges of the box), minimum and maximum values (whiskers), and outliers (open circles)

# **Section 11 – Test of assumptions of the LMM and GLMMs**

For the statistical analyses of whether *B. afzelii* infection and egg washing treatment influenced each of the five immature tick life history traits, we tested the assumptions of the linear mixed effects models (LMMs) and the generalized linear mixed effects models (GLMMs). LMMs assume that the residuals follow a normal distribution and that the variance of the residuals is the same over the range of predicted values (or between groups). We used the Shapiro-Wilk normality test to test the assumption of normality for the residuals. We used a Bartlett’s K-squared test to test whether the variance of the residuals was the same between the four groups, which are as follows: (i) eggs washed with water + larvae fed on uninfected control mice, (ii) eggs washed with water + larvae fed on *B. afzelii*-infected mice, (iii) eggs washed with bleach + larvae fed on uninfected control mice, and (iv) eggs washed with bleach + larvae fed on *B. afzelii*-infected mice. GLMMs assume that the ratio of the residual deviance to the residual degrees of freedom is ~1. If the ratio is greater than 1, the data is said to be overdispersed, which can result in Type I errors. In contrast, if the ratio is less than 1, the data is said to be underdispersed and no further correction is needed.

For immature tick survival, the ratio of the residual deviance (1470.0) to the residual degrees of freedom (1734) was 0.848, which is less than 1.000. Hence, the residuals of immature tick survival met the assumption of the GLMM and no further correction is needed.

For larva-to-nymph moulting success, the ratio of the residual deviance (951.7) to the residual degrees of freedom (1734) was 0.549, which is less than 1.000. Hence, the residuals of larva-to-nymph moulting success met the assumption of the GLMM and no further correction is needed.

For the log10-transformed engorged larval weight (n = 742), the Shapiro-Wilk test found that the residuals were not normally distributed (W = 0.97229, p = 1.203e-10). However, this test is notoriously sensitive to outliers. After excluding 9 negative residuals that were furthest away from the mean, the residuals followed a normal distribution (W = 0.99603, p = 0.061). The Bartlett’s K-squared test found that the variance of the log10-transformed engorged larval weights was the same between the four groups (Bartlett’s K-squared = 6.3322, df = 3, p = 0.097). It is unlikely that these minor deviations from normality would have biased the statistical analysis of the log10-transformed engorged larval weight.

For the log10-transformed unfed nymphal weight (n = 1083), the Shapiro-Wilk test found that the residuals were not normally distributed (W = 0.98414, p = 1.794e-09). However, after excluding 10 negative residuals that were furthest away from the mean, the residuals followed a normal distribution (W = 0.99787, p = 0.185). The Bartlett’s K-squared test found that the variance of the log10-transformed unfed nymphal weights was the same between the four groups (Bartlett’s K-squared = 2.62, df = 3, p-value = 0.454). It is unlikely that these minor deviations from normality would have biased the statistical analysis of the log10-transformed unfed nymphal weight.

For the larva-to-nymph moulting time (n = 1529), the Shapiro-Wilk test found that the residuals were not normally distributed (W = 0.93111, p < 2.2e-16). The Bartlett’s K-squared test found that the variance of the larva-to-nymph moulting times was significantly different between the four groups (Bartlett’s K-squared = 16.604, df = 3, p-value = 0.0008524). To deal with the fact that the residuals violated the assumptions of the LMM, we used an independent two-samples Wilcoxon test to determine whether mouse infection status had a significant effect on the larva-to-nymph moulting time. This test confirmed that the effect of mouse infection status on the larva-to-nymph moulting time was highly significant (W = 359542, p = 0.00000722).

# **Section 12 – Effects of tick family and mouse ID on the life history traits of immature *I. ricinus* ticks**

We found significant effects of mouse ID and tick family on all five life history traits, except for the effect of tick family on larva-to-nymph moulting success (**Table S4**). For the engorged larval weight (log10-transformed), tick family, mouse ID, and the residuals accounted for 8.0%, 5.8%, and 86.3% of the variance, respectively (**Table S5**). Similarly, for the unfed nymphal weight (log10-transformed), tick family, mouse ID, and the residuals accounted for 7.9%, 4.0% and 88.1% of the variance, respectively (**Table S5**). Thus, most of the variance in immature tick weight occurred at the level of the individual ticks and remains unexplained.

**Table S4.** Statistical analyses of the five life history traits of the immature *I. ricinus* ticks. The five life history traits include engorged larval weight, unfed nymphal weight, larva-to-nymph survival, larva-to-nymph moulting success, and larva-to-nymph moulting time. A variety of models (LMs, GLMs, LMMs, and GLMMs) were used to test the effects of tick family and mouse ID on the five response variables. Shown are the results from the type II log-likelihood ratio test to determine the statistical significance of the explanatory variables. The row headers refer to the name of the response variable, whether infection status was determined by the mouse or the tick, the name of the explanatory variable, the degrees of freedom, the Chi-square statistic, and the p-value.

| **Variable** | **Factor** | **Model** | **Df** | **χ^2^ or F** | **p** |
| --- | --- | --- | --- | --- | --- |
| Engorged larval weight | Family | LMM | 9 | 34.314 | <0.001 |
| Engorged larval weight | Mouse | LM | 37, 704 | F = 3.9347 | <0.001 |
|  |  |  |  |  |  |
| Unfed nymphal weight | Family | LMM | 9 | 23.873 | 0.005 |
| Unfed nymphal weight | Mouse | LM | 38, 1044 | F = 4.5098 | <0.001 |
|  |  |  |  |  |  |
| Survival | Family | GLMM | 9 | 19.701 | 0.020 |
| Survival | Mouse | GLM | 38 | 99.571 | <0.001 |
|  |  |  |  |  |  |
| Moulting success | Family | GLMM | 9 | 8.1931 | 0.515 |
| Moulting success | Mouse | GLM | 38 | 101.22 | <0.001 |
|  |  |  |  |  |  |
| Moulting time | Family | LMM | 9 | 80.647 | <0.001 |
| Moulting time | Mouse | LM | 38, 1559 | F = 4.6287 | <0.001 |

**Table S5.** Variance in the log10-transformed weights of the engorged larvae and the unfed nymphs (originally measured in μg). The variance components were estimated from a linear mixed effects model where tick family and mouse ID were modelled as random effects. The variance components were converted to percentages.

| **Stage** | **Source of Variation** | **Variance (x 10^3)** | **% Variance** |
| --- | --- | --- | --- |
| Engorged larva | Tick Family | 0.3014 | 8.0 |
| Engorged larva | Mouse ID | 0.2179 | 5.8 |
| Engorged larva | Residual | 3.2647 | 86.3 |
|  |  |  |  |
| Unfed nymph | Tick Family | 0.3204 | 7.9 |
| Unfed nymph | Mouse ID | 0.1606 | 4.0 |
| Unfed nymph | Residual | 3.553 | 88.1 |

# **Section 13 – Power analysis to determine the minimal detectable effect size**

For each of the five life history traits of the immature *I. ricinus* ticks, we conducted a power analysis to determine the effect size that we could have detected with our sampling effort. We used the *powerSim()* function in the simr package to determine the power for a range of effect sizes. We investigated a range of effect sizes where the *B. afzelii* infection treatment either increased or decreased the tick phenotype compared to the control group. We did not conduct power analyses for the effect of non-pathogenic tick-associated bacteria on tick phenotype, as this would have given similar results to the power analyses for the *B. afzelii* infection treatment. For each of the five life history traits of the immature *I. ricinus* ticks, we created power curves by graphing the power versus the effect size. The effect sizes were expressed as follows: the mean phenotype of the ticks that were in the infected group were calculated as a percentage of the mean phenotype of the ticks in the control group. **Figures S7 to S11** show the results of the power analyses for the five life history traits of the immature *I. ricinus* ticks.

**Figure S7.** Power curve for the experimental design to detect a significant effect of *B. afzelii* infection on survival of immature *I. ricinus* ticks. The effect size was expressed as follows: the mean phenotype of the ticks that were in the infected group were calculated as a percentage of the mean phenotype of the ticks in the control group. For a power threshold of 80%, our sampling effort had the ability to detect the following effect sizes of *B. afzelii* infection on the survival of immature *I. ricinus* ticks: a reduction of ~9% and an increase of 7%.

**Figure S8.** Power curve for the experimental design to detect a significant effect of *B. afzelii* infection on survival of larva-to-nymph moulting success of *I. ricinus* ticks. The effect size was expressed as follows: the mean phenotype of the ticks that were in the infected group were calculated as a percentage of the mean phenotype of the ticks in the control group. For a power threshold of 80%, our sampling effort had the ability to detect the following effect sizes of *B. afzelii* infection on the larva-to-nymph moulting success of *I. ricinus* ticks: a reduction of ~7% and an increase of 4%.

**Figure S9.** Power curve for the experimental design to detect a significant effect of *B. afzelii* infection on larva-to-nymph moulting time of *I. ricinus* ticks. The effect size was expressed as follows: the mean phenotype of the ticks that were in the infected group were calculated as a percentage of the mean phenotype of the ticks in the control group. For a power threshold of 80%, our sampling effort had the ability to detect the following effect sizes of *B. afzelii* infection on the larva-to-nymph moulting time of *I. ricinus* ticks: a reduction of ~6% and an increase of 6%.

**Figure S10.** Power curve for the experimental design to detect a significant effect of *B. afzelii* infection on weight of engorged *I. ricinus* larvae. The effect size was expressed as follows: the mean phenotype of the ticks that were in the infected group were calculated as a percentage of the mean phenotype of the ticks in the control group. For a power threshold of 80%, our sampling effort had the ability to detect the following effect sizes of *B. afzelii* infection on the weight of engorged *I. ricinus* larvae: a reduction of ~4% and an increase of 4%.

**Figure S11.** Power curve for the experimental design to detect a significant effect of *B. afzelii* infection on weight of unfed *I. ricinus* nymphs. The effect size was expressed as follows: the mean phenotype of the ticks that were in the infected group were calculated as a percentage of the mean phenotype of the ticks in the control group. For a power threshold of 80%, our sampling effort had the ability to detect the following effect sizes of *B. afzelii* infection on the weight of unfed *I. ricinus* nymphs: a reduction of ~4% and an increase of 4%.

References

Bakke, I., P. De Schryver, N. Boon, and O. Vadstein. 2011. PCR-based community structure studies of Bacteria associated with eukaryotic organisms: A simple PCR strategy to avoid co-amplification of eukaryotic DNA. Journal of Microbiological Methods **84**:349-351.

Belli, A., A. Sarr, O. Rais, R. O. M. Rego, and M. J. Voordouw. 2017. Ticks infected via co-feeding transmission can transmit Lyme borreliosis to vertebrate hosts. Scientific Reports **7**:1-13.

Bistolas, K. S. I., R. I. Sakamoto, J. A. M. Fernandes, and S. K. Goffredi. 2014. Symbiont polyphyly, co-evolution, and necessity in pentatomid stinkbugs from Costa Rica. Frontiers in Microbiology **5**.

Bueche, M., T. Wunderlin, L. Roussel-Delif, T. Junier, L. Sauvain, N. Jeanneret, and P. Junier. 2013. Quantification of endospore-forming Firmicutes by quantitative PCR with the functional gene *spo0A*. Applied and Environmental Microbiology **79**:5302-5312.

Couret, J., M. C. Dyer, T. N. Mather, S. Han, J. I. Tsao, R. A. Lebrun, and H. S. Ginsberg. 2017. Acquisition of *Borrelia burgdorferi* infection by larval *Ixodes scapularis* (Acari: Ixodidae) associated with engorgement measures. Journal of Medical Entomology **54**:1055-1060.

Muyzer, G., E. C. Dewaal, and A. G. Uitterlinden. 1993. Profiling of complex microbial populations by denaturing gradient gel electrophoresis analysis of polymerase chain reaction-amplified genes coding for 16s ribosomal RNA. Applied and Environmental Microbiology **59**:695-700.

Prado, S. S., and R. P. P. Almeida. 2009. Role of symbiotic gut bacteria in the development of *Acrosternum hilare* and *Murgantia histrionica*. Entomologia Experimentalis et Applicata **132**:21-29.

Prado, S. S., D. Rubinoff, and R. P. P. Almeida. 2006. Vertical transmission of a pentatomid caeca-associated symbiont. Annals of the Entomological Society of America **99**:577-585.

Salem, H., E. Kreutzer, S. Sudakaran, and M. Kaltenpoth. 2013. Actinobacteria as essential symbionts in firebugs and cotton stainers (Hemiptera, Pyrrhocoridae). Environmental Microbiology **15**:1956-1968.

Sassera, D., N. Lo, E. A. P. Bouman, S. Epis, M. Mortarino, and C. Bandi. 2008. "Candidatus Midichloria" endosymbionts bloom after the blood meal of the host, the hard tick Ixodes ricinus. Applied and Environmental Microbiology **74**:6138-6140.

Schwaiger, M., O. Peter, and P. Cassinotti. 2001. Routine diagnosis of *Borrelia burgdorferi* (sensu lato) infections using a real-time PCR assay. Clinical Microbiology and Infection **7**:461-469.

Taylor, C. M., P. L. Coffey, B. D. DeLay, and G. P. Dively. 2014. The importance of gut symbionts in the development of the brown marmorated stink bug, *Halyomorpha halys* (Stal). PLOS ONE **9**.
